# PredGCN: a Pruning-enabled Gene-Cell Net for automatic cell annotation of single cell transcriptome data

**DOI:** 10.1093/bioinformatics/btae421

**Published:** 2024-06-26

**Authors:** Qi Qi, Yunhe Wang, Yujian Huang, Yi Fan, Xiangtao Li

**Affiliations:** School of Artificial Intelligence, Jilin University, Changchun 130012, China; School of Artificial Intelligence, Hebei University of Technology, Tianjin 300401, China; College of Computer Science and Cyber Security, Chengdu University of Technology, Chengdu 610059, China; School of Artificial Intelligence, Jilin University, Changchun 130012, China; School of Artificial Intelligence, Jilin University, Changchun 130012, China

## Abstract

**Motivation:**

The annotation of cell types from single-cell transcriptomics is essential for understanding the biological identity and functionality of cellular populations. Although manual annotation remains the gold standard, the advent of automatic pipelines has become crucial for scalable, unbiased, and cost-effective annotations. Nonetheless, the effectiveness of these automatic methods, particularly those employing deep learning, significantly depends on the architecture of the classifier and the quality and diversity of the training datasets.

**Results:**

To address these limitations, we present a Pruning-enabled Gene-Cell Net (PredGCN) incorporating a Coupled Gene-Cell Net (CGCN) to enable representation learning and information storage. PredGCN integrates a Gene Splicing Net (GSN) and a Cell Stratification Net (CSN), employing a pruning operation (PrO) to dynamically tackle the complexity of heterogeneous cell identification. Among them, GSN leverages multiple statistical and hypothesis-driven feature extraction methods to selectively assemble genes with specificity for scRNA-seq data while CSN unifies elements based on diverse region demarcation principles, exploiting the representations from GSN and precise identification from different regional homogeneity perspectives. Furthermore, we develop a multi-objective Pareto pruning operation (Pareto PrO) to expand the dynamic capabilities of CGCN, optimizing the sub-network structure for accurate cell type annotation. Multiple comparison experiments on real scRNA-seq datasets from various species have demonstrated that PredGCN surpasses existing state-of-the-art methods, including its scalability to cross-species datasets. Moreover, PredGCN can uncover unknown cell types and provide functional genomic analysis by quantifying the influence of genes on cell clusters, bringing new insights into cell type identification and characterizing scRNA-seq data from different perspectives.

**Availability and implementation:**

The source code is available at https://github.com/IrisQi7/PredGCN and test data is available at https://figshare.com/articles/dataset/PredGCN/25251163.

## 1 Introduction

The development of single-cell sequencing technologies has unveiled the heterogeneity and intricacies of RNA transcripts at the cellular level (Jovic *et al.* 2022, Fan *et al.* 2023). Unlike traditional bulk RNA sequencing, which averages expression across cell populations, single-cell RNA sequencing (scRNA-seq) technology can identify the RNA transcriptional landscapes of each cell and quantify the expression of genes that can be detected, revealing the stochastic nature of gene expression in higher resolution (Watanabe *et al.* 2019, Kashima *et al.* 2020, Su *et al.* 2024). A frequent task involves identifying and characterizing cell types for downstream analysis, as the variety and ratio of cell types illuminate the functionality and condition of tissues or organs (Yu *et al.* 2023). Typically, this involves unsupervised clustering of the dataset, followed by manual annotation of cell types using cluster results and knowledge of marker genes. However, the number of cell types is not known in advance can introduce bias into the clustering, and manual annotation is time-consuming, labor-intensive and subjective, which can affect the reliability of the results.

As the advance of cell type marker databases such as CellMarker (Zhang *et al.* 2019b) and PanglaoDB (Franzén *et al.* 2019), automatic annotation methods for the cell types of scRNA-seq clusters have been introduced; for instance, CellAssign (Zhang *et al.* 2019a) leveraged prior knowledge of cell-marker genes by a probabilistic model to identify cell types. SCINA (Zhang *et al.* 2019c) used an expectation-maximization algorithm to exploit previously known gene markers. scCATCH (Shao *et al.* 2020) annotated cell types through the tissue-specific cellular taxonomy reference database and the evidence-based scoring protocol. However, challenges arise when marker genes are associated with multiple cell types or when cell types lack known markers, and the construction of marker-gene sets is still labor-intensive and subjective, leading to potential deviations in annotation results.

Beyond marker-gene datasets, numerous scRNA-seq datasets with reliably labeled cell types exist, leading to the development of cell-based annotation methods. These methods determine potential cell annotations by comparing query scRNA-seq datasets with reference datasets. They fall into two categories: correlation-based and supervised annotation methods (Pasquini *et al.* 2021). Correlation-based annotation methods, such as scmap (Kiselev *et al.* 2018), SingleR (Aran *et al.* 2019), and CHETAH (De Kanter *et al.* 2019), use a reference dataset to reveal information about the query dataset by comparing gene expression levels to evaluate the similarities between datasets. Supervised annotation methods, such as CaSTLe (Lieberman *et al.* 2018), SingleCellNet (Tan and Cahan 2019), scPred (Alquicira-Hernandez *et al.* 2019) and ACTINN (Ma and Pellegrini 2020), apply machine learning to transfer labels from reference data to query data. They use the annotated reference data to build a model distribution of cell types and then apply the trained model to annotate query data according to their relative features. While these tools offer valuable insights, it’s evident that current supervised annotation methods heavily rely on specific machine learning models, such as neural network architectures or algorithms. This dependency often results in performance closely linked to the chosen model, limiting effectiveness across diverse classifiers or datasets. Furthermore, these methods are susceptible to overfitting, particularly with high-dimensional sparse data, leading to inadequate generalization to new datasets.

To overcome these challenges and facilitate scalable annotation, we have developed PredGCN, a novel framework that integrates diverse computational techniques to form a multidirectional coverage search network. This network efficiently synthesizes information from reference datasets and employs a pruning strategy to optimize the network configuration for specific scenarios, thereby enhancing both performance and adaptability. Experiments validate PredGCN’s superior performance over comparative approaches in accuracy across benchmark scRNA-seq datasets and demonstrate remarkable scalability and extensibility in cross-species dataset evaluations. Furthermore, PredGCN has the capability to identify cell types not present in the reference dataset and performs functional genomic analysis to evaluate the influence of genes on unique cell clusters, thus providing novel insights into cell type identification and a comprehensive characterization of scRNA-seq data. Additionally, a user-friendly webserver has been developed to facilitate the application of PredGCN.

## 2 Materials and methods

### 2.1 Methodology overview of PredGCN

**Figure 1.**
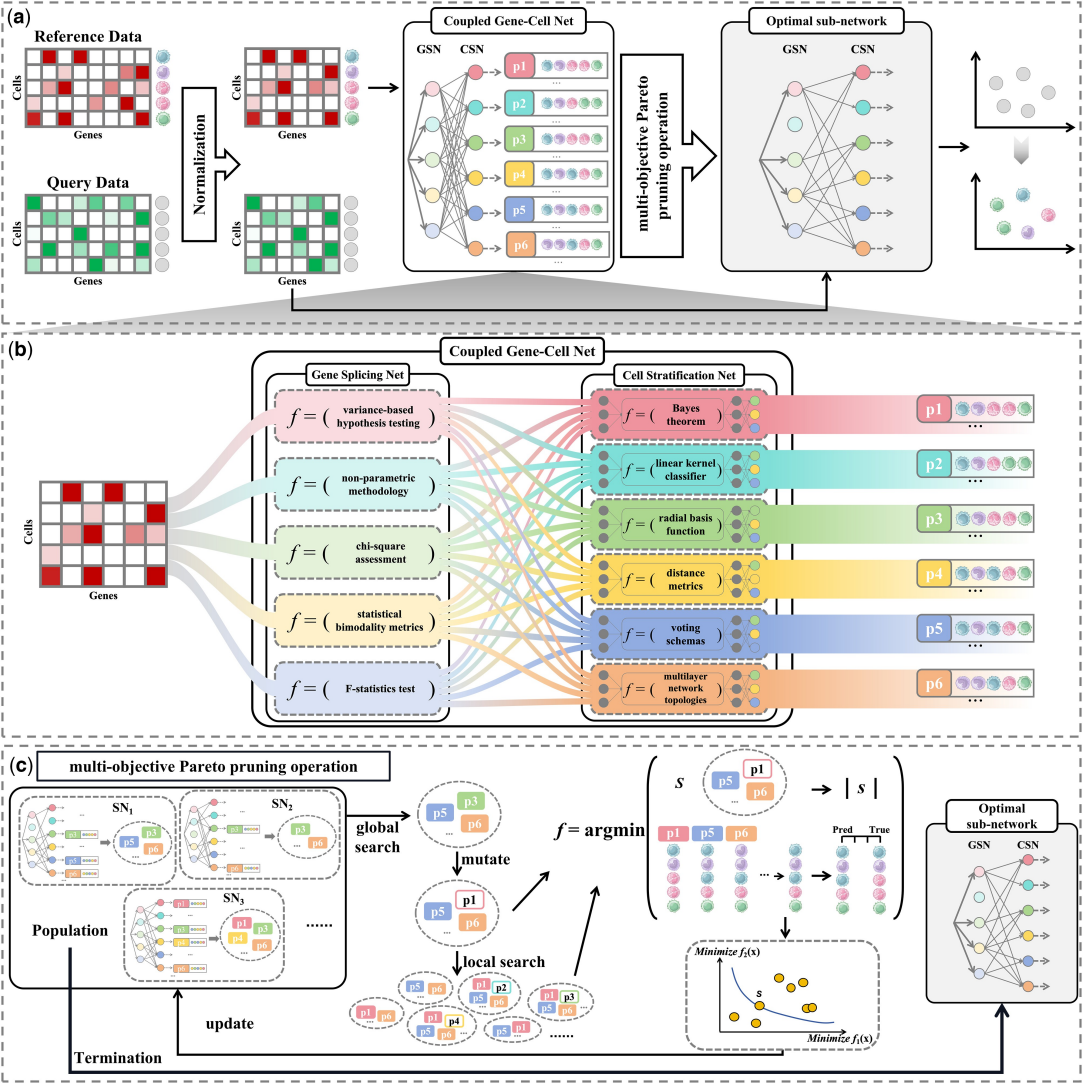
Overview of the proposed method: PredGCN for Automatic cell Annotation of Single Cell Transcriptome Data. (a) Framework of PredGCN. The PredGCN framework begins with the normalization of both reference and query data. Post-normalization, the reference data are utilized to train a classifier known as the Coupled Gene-Cell Net (CGCN). This network is subsequently processed using the multi-objective Pareto Pruning Operation (PrO) to derive the optimal sub-network. This optimal sub-network is then used to annotate the query data. (b) Framework of CGCN. The CGCN framework facilitates representation learning and information storage by training a CGCN that integrates a Gene Splicing Net (GSN) and a Cell Stratification Net (CSN). The GSN identifies significant genes from complementary perspectives using diverse statistical and hypothesis-testing-based feature selection nodes. Concurrently, the CSN fully exploits the representations from the GSN through various region delineation principles, ensuring comprehensive utilization of the gene selection information. (c) Framework of multi-objective Pareto Pruning Operation. This framework includes steps for population initialization, global search, local search, the objective function, and termination. In each iteration, a global search is first performed to ensure that all sub-networks have the opportunity to be explored. The sub-network identified from the global search are then subjected to a local search, where distinct CGCN frameworks are generated by greedily searching the neighborhoods of mutated sub-networks to improve the quality of new candidate sub-networks, thereby increasing efficiency. The objective function manages the intrinsic complexity of comparing multiple objectives using domination relationships and updates the population during both global and local searches. Upon meeting the termination condition, the optimal sub-network is identified.

PredGCN, tailored for scRNA-seq data, leverages transcriptome references to pinpoint unknown cell types. The framework of PredGCN is composed of four principal components, as illustrated in Fig. 1. (i) The process initiates with normalization of the reference and query data to ensure uniform expression levels across genes and cells. (ii) A Coupled Gene-Cell Net (CGCN) linking a Gene Splicing Net (GSN) and a Cell Stratification Net (CSN) is trained using the normalized reference data. The GSN employs diverse statistical methods and hypothesis-testing principles to identify significant genes from complementary perspectives. These identified genes are then linked to the CSN, which utilizes principles of heterogeneous region demarcation to establish a foundational framework for the identification results. (iii) The subsequent phase involves a multi-objective Pareto pruning operation (Pareto PrO) to refine CGCN, focusing on the most impactful sub-network for the analysis. (iv) The final stage achieves cell type identification in the query data through a consensus operation on the pruned sub-network’s results, ensuring precise and comprehensive cell type annotation.

### 2.2 Data collection and preprocessing

To comprehensively evaluate PredGCN across various contexts, we curated twelve real scRNA-seq datasets spanning different species (human and mouse), organs (pancreas and PBMC), and sequencing platforms (including SMARTer, 10× Genomics, inDrop, CEL-seq2, Smart-seq2, Drop-seq and Seq-well). Specifically, the human pancreas datasets from Muraro (Muraro *et al.* 2016), Segerstolpe (Segerstolpe *et al.* 2016), and Xin (Xin *et al.* 2016) are denoted as hMuraro, hSegerstolpe and hXin, respectively. The Baron (Baron *et al.* 2016) dataset contains both human and mouse pancreas data, denoted as hBaron and mBaron, respectively. These datasets enable to assess the performance of PredGCN in both same-species and cross-species scenarios. Additionally, we leveraged healthy and systemic lupus erythematosus (SLE) PBMC datasets to validate the capacity of PredGCN to elucidate potential disease signals and therapeutic targets. Further details on these datasets are available in the Supplementary Section 12.

PredGCN inputs an scRNA-seq count matrix ***C*** for ***n*** cells, ***m*** genes, and ***k*** cell types. Since the inherent noise in scRNA-seq data, quality control involves filtering out exceptionally sparsely expressed genes and cells. Subsequently, the matrix ***C*** is normalization by dividing each row by its sum and scaling by the median library size across cells, followed by a log-transformation to yield continuous values. This adjustment ameliorates systemic variations to achieve standardized expression measures across genes and cells. After that, the normalized scRNA-seq count matrix is denoted as $M_{i}^{j},i=\left\{1,2,\ldots ,\bar{n}\right\},j=\left\{1,2,\ldots ,\bar{m}\right\}$.

### 2.3 Coupled Gene-Cell Net

To address the diversity in scRNA-seq datasets, a Coupled Gene-Cell Net (CGCN) is developed for representation learning and information storage. This network combines a Gene Splicing Net (GSN), which uses statistical methods and hypothesis testing to identify significant genes, with a Cell Stratification Net (CSN) that employs different region delineation strategies for diverse identification outcomes. The CSN utilizes genes identified by GSN, ensuring that the network captures and utilizes gene-specific nuances for accurate cell type identification.

#### 2.3.1 Gene Splicing Net

Individual feature selection methods harbor intrinsic biases, while concatenating multiple approaches can coalesce diverse vantages for optimizing interpretation of insightful genes (Bolón-Canedo and Alonso-Betanzos 2019). Furthermore, retaining predictive albeit redundant transcripts is indispensable for precision representation of the multifaceted heterogeneity inherent across cell states. Therefore, we construct a Gene Splicing Net (GSN) which synergistic five discrete feature extraction modalities to selectively assemble both discriminative and integral redundant genes. One node of the GSN resorts variance-based hypothesis testing to actualize feature selection by evaluating inter-gene correlation structures. It can be denoted as:

(1) $$T=\frac{\left(\bar{n}-1\cdot \ln(W)\right)}{df}$$

where *W* is the ratio of the variance of each feature. *df* is the degrees of freedom, used to adjust the size of the statistic. Implementing a non-parametric methodology, an additional node pursues feature selection by assessing distributional divergences across featured dimensions underlying cell state space. It can be denoted by the formula:

(2) $$T=\max|F_{y}-F_{x}|$$

where *F_x_* and *F_y_* are the empirical distribution functions of the cell type *x* and of all the other cell types *y*. Via chi-square assessments of correlational patterns between categorical features, another modular GSN node enacts feature filtering based on stochastic affinity. It is denoted as:

(3) $$T=\frac{{\left(O_{x}-E_{x}\right)}^{2}}{E_{x}}+\frac{{\left(O_{y}-E_{y}\right)}^{2}}{E_{y}}$$

where *O_x_* and *O_y_* are the number of observations of *x* and *y*. *E_x_* and *E_y_* are the number of expectations in the 2-dimensional contingency table of *x* and *y*. Through computing statistical bimodality metrics across features, an auxiliary GSN node achieves feature selection by capitalizing on modality-derived signals. It can be expressed as:

(4) $$T=\frac{\left|{\text{mean}}_{x}-{\text{mean}}_{y}\right|}{p_{x}\times s_{x}+p_{y}\times s_{y}}$$

where mean_*x*_ and mean_*y*_ are the means of the expression levels of the cell type *x* and of all the other cell types *y*, *s* is the standard deviation, and *p* is the proportion of cells of the cell type under consideration. An auxiliary node utilizes *F*-statistics to realize feature filtering by evaluating variance contrasts between feature subsets (Aran *et al.* 2019, Su *et al.* 2021). The *F*-statistics test for the gene *g* is expressed as:

(5) $$F_{g}=\frac{\mathrm{var}b_{g}/df_{1}}{(\mathrm{var}t_{g}-var_{b})/df_{2}}$$

where *df*_1_ and *df*_2_ are degrees of freedoms. *vart_g_* is the total between variance and *varb_g_* is the between-group variance. More details about GSN are provided in the Supplementary Material.

#### 2.3.2 Cell stratification net

Building on the GSN, we subsequently construct modular nodes for orchestrating cell type identification, termed as Cell Stratification Net (CSN). Given the assumptions intrinsic to machine learning models limit single classifiers for fully utilizing multifaceted features, the CSN brings together multiple nodes based on various region delineation principles to fully capitalize on the representations of the GSN. A node within the CSN operates under Bayes’ theorem assumptions to categorize cells into putative identities based on statistical analytics of transcriptional profiles. The classification process can be expressed as:

(6) $$\hat{y}=\arg\underset{y_{k}}{\max}(P(y_{k})\times \begin{array}{c} \prod_{j=1}^{\bar{m}}P(M_{i}^{j}|y_{k}) \end{array})$$

where $P(y_{k})$ is the prior probability of the cell type *y_k_*. Seeking to discern the optimal linear boundary demarcating dataset partitions, the second node of the CSN operates as a linear kernel classifier. This constituent constructs *k* hyperplanes for *k*-class problem. The *i*th hyperplane is trained with the cells between the *i*th type and other types. The process of label identification can be expressed as:

(7) $$\hat{y}=\arg\underset{y_{k}}{\max}(f_{y_{k}}(M_{i}))$$

where $f_{y_{k}}(M_{i})$ is the score of cell *M_i_* belonging to type *y_k_*. The third node conductor leverages the radial basis function to project input space onto a higher-dimensional feature space, where the classification task is facilitated by identifying the optimal separating hyperplane. The identification process can be expressed as:

(8) $$\hat{y}=\arg\underset{y_{k}}{\max}(\sum_{j\neq k}h_{jk}(M_{i}))$$

where *h_jk_* denotes the classifier between type *y_k_* and type *y_j_*. Employing distance metrics to gauge inter-cellular similarities, the fourth node provides an additional signal for delineating cell identity boundaries. For cell *M_i_* and the *k* cells closest to it, the prediction process can be denoted as:

(9) $$\hat{y}=\arg\underset{y_{j}}{\max}(\mathrm{freq}(y_{j}))$$

where $\mathrm{freq}(y_{j})$ is the frequency of type *y_j_* in the *k* nearest neighbor cells. Through aggregating modular outcomes via averaging or plurality voting schemas, the fifth node condenses multilayered predictions to enhance consensus. The type of cell *M_i_* is denoted as:

(10) $$\hat{y}=\arg\underset{y_{k}}{\max}(h_{y_{k}}(M_{i}))$$

where $h_{y_{k}}$ is the probability of predicting for type *y_k_*. Embedding nonlinear activation operations upon multilayer network topologies, the sixth constituent captures higher-order feature combinations to complement partial profile perspectives. The process of identifying cell *M_i_* with *L* layer network and *ReLU* activation can be denoted as:

(11) $$\hat{y}=\arg\underset{y_{k}}{\max}(\sigma (h_{y_{k}}(M_{i})))$$

where $\sigma (h_{y_{k}}(M_{i})$ denotes the predicted probability that cell *M_i_* belongs to type *y_k_*. σ(·) is the activation function of the output layer.

### 2.4 Pruning operation

In order to enable CGCN to dynamically face the multiple complexity challenges in heterogeneous cell recognition, we design a multi-objective Pareto Pruning Operation (Pareto PrO). The multi-objective Pareto PrO automatically seeks the optimal architecture for the CGCN, and consists of five important components: population initialization, global search, local search, the objective function, and termination.

#### 2.4.1 Population initialization

The *l* identification paths in CGCN are formed by connecting nodes within the GSN and CSN, and each path generates a distinguishable identification result. By harmonizing the recognition results of several outstanding paths, the complementarity between paths can improve the identification ability of the CGCN, as well as subtract the redundant paths to optimize the structure of CGCN. To find an optimal sub-network structure consisting of multiple interconnected paths, we design a multi-objective Pareto PrO. The sub-network is represented by a *l*-dimensional binary vector:

(12) $$s\in {\left\{0,1\right\}}^{l}$$

with 0, 1 indicating that the corresponding path is absent or in the sub-network, respectively. $N_{s}$ denotes the set of identification results of paths in sub-network ***s***. The population *Pop* keeps all the candidate sub-networks and initialized with a random candidate sub-network $s_{r}$.

#### 2.4.2 Global search

The global search extracts the optimal candidate sub-network over the entire range of candidate sub-networks. It is described as follows:

(13) $$N_{s\prime }\leftarrow \{\begin{array}{cc} P(N_{s\prime i}=0)=1/q & \text{if}\;N_{si}=1\\ P(N_{s\prime i}=1)=1/q & \text{if}\;N_{si}=0 \end{array}$$

(14) $$\mathrm{Pop}=(\mathrm{Pop}-\{N_{t}\in \mathrm{Pop}|N_{s\prime }{≽}_{f}N_{t}\})\cup N_{s\prime }$$

$N_{s}$ is a random sub-network selected from the *Pop*, and $N_{s\prime }$ is the mutation sub-network generated by $N_{s}$ according to Equation (13). Equation (14) uses the $N_{s\prime }$ to update *Pop*. This global update rule ensures that all sub-networks have the possibility to be searched.

#### 2.4.3 Local search

The local search is performed on the mutation sub-network $N_{s\prime }$. Potential candidate sub-networks are found to update *Pop* by searching on the neighborhood of $N_{s\prime }$. *L* keeps the center of the current local search and initialized as $L=N_{s\prime }$. The local search process is expressed as:

(15) $$V=\{N_{y}|D(L,N_{y})\leq d\}$$

(16) $$Q=Q\cup \{V_{s}|V_{s}\in V\;\text{with the minimal}\;f_{1}\;\text{value}\}$$

(17) $$R=R\cup \{i|V_{si}\neq N_{s\prime i}\}$$

(18) $$L=V_{s}$$

*D* is the Hamming distance and *d* is set to 1 in Equation (15). The *Q* and *R* are used to record the optimal candidate sub-networks that have been searched and the neighborhoods that have been searched, respectively. The local search process stops while V=∅. For $N_{q}\in Q$, *Pop* is updated as:

(19) $$\mathrm{Pop}=(\mathrm{Pop}-\{N_{t}\in \mathrm{Pop}|N_{q}\;{≽}_{f}\;N_{t}\})\cup N_{q}$$

Subsequently, iterate through the aforementioned global search and local search steps until the termination.

#### 2.4.4 Objective function

For a candidate sub-network ***s***, the first objective represents the generalized performance of the sub-network and can be formulated as:

(20) $$E_{s}\leftarrow \{W|w_{i}=\text{mode}(N_{si})\}$$

(21) $$f_{1}=f(N_{s})=\sum_{i=1}^{\bar{n}}I(E_{si}\neq y_{i})$$

where $E_{s}$ is the consensus result of paths in ***s*** and ***y*** indicates the true labels of reference. The second function measures the number of paths in ***s*** and is expressed as:

(22) $$f_{2}=|s|$$

Solving the bi-objective set pruning problem can be expressed by the formulation:

(23) $${\text{arg min}}_{s\in {\left\{0,1\right\}}^{l}}(f_{1},f_{2})$$

The concept of the domination relationship is adopted to cope with the intrinsic complexity of directly comparing more than one objective function. For two candidate sub-networks $s_{1}$ and $s_{2}$, the relation $s_{1}$ weakly dominates $s_{2}$ is denoted as:

(24) $$s_{1}{≽}_{f}\;s_{2}$$

(25) $$\text{if}\;f_{1}(s_{1})\leq f_{1}(s_{2})\;\text{and}\;f_{2}(s_{1})\leq f_{2}(s_{2})$$

Further, the relation $s_{1}$ dominates $s_{2}$ is denoted as:

(26) $$s_{1}{≻}_{f}\;s_{2}$$

(27) $$\text{if}\;f_{1}(s_{1})<f_{1}(s_{2})\;\text{and}\;f_{2}(s_{1})<f_{2}(s_{2})$$

The domination relationship is applied throughout the search process.

#### 2.4.5 Termination

The pruning process concludes once a preset maximum iteration count is reached, selecting the sub-network with the lowest *f*_1_ score from the population *Pop* as optimal. For the query dataset, this optimal sub-network generates a set of identification results, $N_{o}$. These results are then aggregated into a consensus identification, *E*, using a specified Equation (20), setting the stage for further detailed analysis.

### 2.5 Webserver

For user convenience, a dedicated PredGCN webserver has been established, hosted on an Ubuntu 18.04 cloud platform. It features a Bootstrap-based user interface and Django for backend operations, ensuring compatibility across browsers and removing hardware or software constraints. The PredGCN web server, developed with Python and R and supported by a MongoDB database for data management, is now publicly accessible at the following URL: http://www.aibio-lab.com/PredGCN/index/. A tutorial for using the PredGCN webserver is available in the Supplementary Sections 11 and 13, guiding users through its features and functionalities to ensure effective utilization.

## 3 Results

### 3.1 Evaluation metrics

To evaluate the quality of cell annotation in PredGCN, we use Accuracy (Acc), F1-score, and Matthews Correlation Coefficient (MCC), comparing predictions to true labels through four categories: True Positives (TP) for correct predictions, False Positives (FP) for misclassified cells, False Negatives (FN) for missed correct classifications, and True Negatives (TN) for accurately identifying non-target cells. The Acc provides an effective metric for evaluating the ability of a method to correctly identify cell types. The F1-score incorporates both precision and recall into a composite measure. The MCC is employed to assess multi-class prediction quality. The complete formula descriptions for these metrics refer to the Supplementary Section 9.

### 3.2 Related methods from the literature

The performance of PredGCN was benchmarked against a range of state-of-the-art algorithms, involving both deep learning models (Yang *et al.* 2021) including graph convolutional network (GCN), deep neural network (DNN), and stacked autoencoder (SAE) and single-cell classification algorithms such as SingleCellNet (Tan and Cahan 2019), SingleR (Aran *et al.* 2019), CaSTLe (Lieberman *et al.* 2018), scmap-cluster and scmap-cell (Kiselev *et al.* 2018). Additionally, we also benchmarked PredGCN against six standalone machine learning classifiers including multinomial Naive Bayes (MNB), Support Vector Machine (SVM), Radial Basis Function Support Vector Machine (rbfsvm), k-nearest neighbors (KNN), random forest (rf), and Multi-layer Perceptron (MLP) on cross-species datasets, revealing PredGCN’s broad applicability and effectiveness in cell type identification. More detailed configurations of those comparison methods are provided in Supplementary Section 10.

### 3.3 Evaluation on real scRNA-seq data

**Figure 2.**
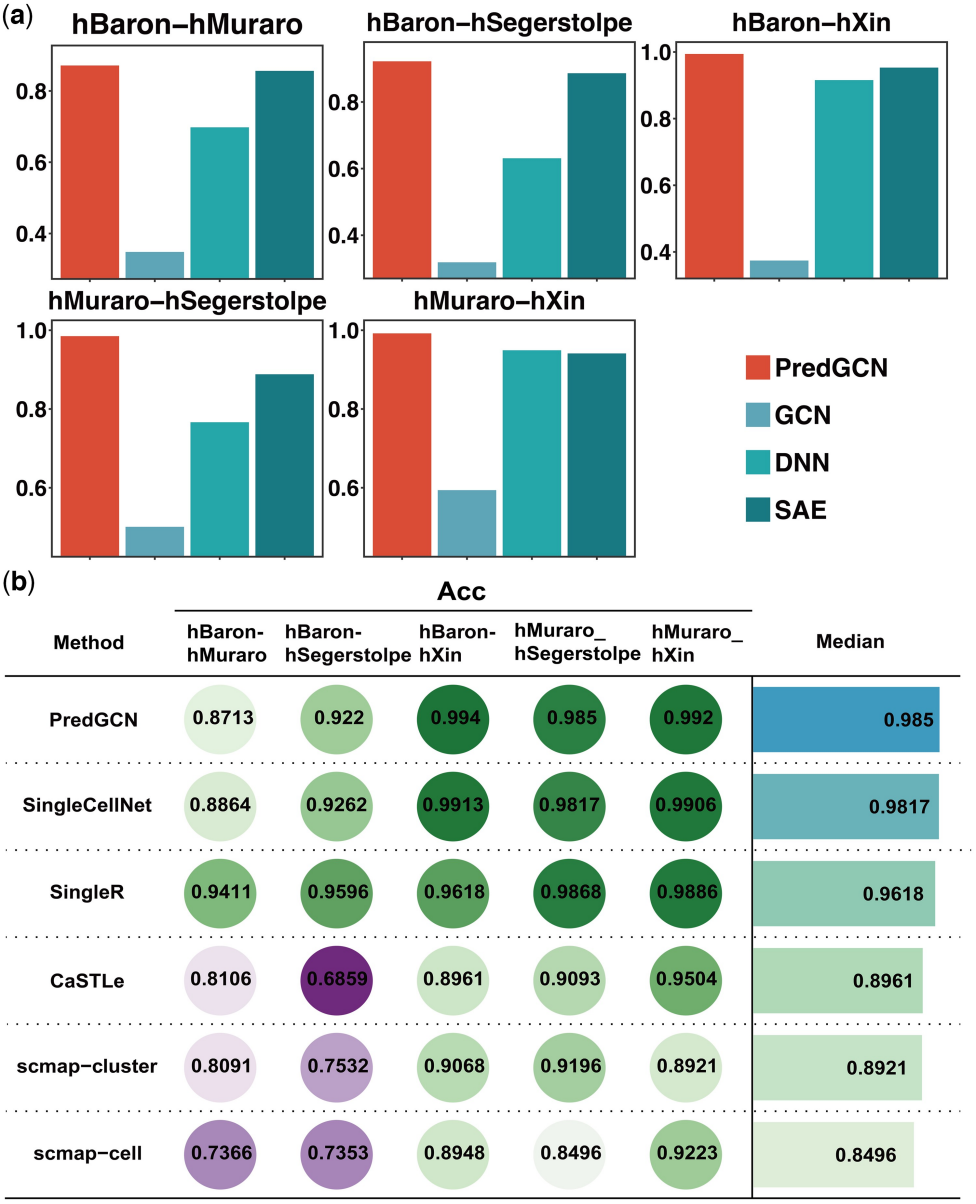
(a) Comparison results for PredGCN and three deep learning algorithms on five real scRNA-seq data measured by Acc. (b) Comparison results for PredGCN and five single cell algorithms on five real scRNA-seq data measured by Acc.

The PredGCN framework was evaluated for its efficacy in analyzing scRNA-seq data against a benchmark comprising three deep learning algorithms (GCN, DNN, SAE) and five single-cell analysis tools (SingleCellNet, SingleR, CaSTLe, scmap-cluster, scmap-cell). This evaluation utilized five real scRNA-seq datasets representing gene expression in the human pancreas, an organ vital for nutrient metabolism regulation. The datasets included hBaron-hMuraro, hBaron-hSegerstolpe, hBaron-hXin, hMuraro-hSegerstolpe, and hMuraro-hXin. Figure 2a shows the Acc metrics obtained by comparing predicted to true cell types. PredGCN achieves the highest Acc values among deep learning methods across all datasets, with notable scores of 0.99 in both hBaron-hXin and hMuraro-hXin datasets, exceeding 0.9 and 0.87 in the hBaron-hSegerstolpe and hBaron-hMuraro datasets, respectively. This superior performance is further corroborated by the F1-score and MCC metrics, detailed in Supplementary Figs S1 and S2. Additionally, Fig. 2b illustrates that PredGCN attained the highest Acc values among all the single-cell algorithms for the hBaron-hXin and hMuraro-hXin datasets. In the case of hBaron-hMuraro and hBaron-hSegerstolpe datasets, SingleR emerged as the most accurate. However, PredGCN, alongside SingleR, recorded the highest and most superior Acc values on the hMuraro-hSegerstolpe dataset, establishing its median Acc as superior compared with those of the competing algorithms. This trend is consistent with the median F1-scores and MCC, as depicted in Supplementary Figs S3 and S4. In summary, the comprehensive benchmarking highlights PredGCN’s competitive advantage, demonstrating its ability to compete with established deep learning algorithms and single-cell algorithms in analyzing homologous scRNA-seq datasets.

### 3.4 Capability of PredGCN in cross-species scenarios

**Figure 3.**
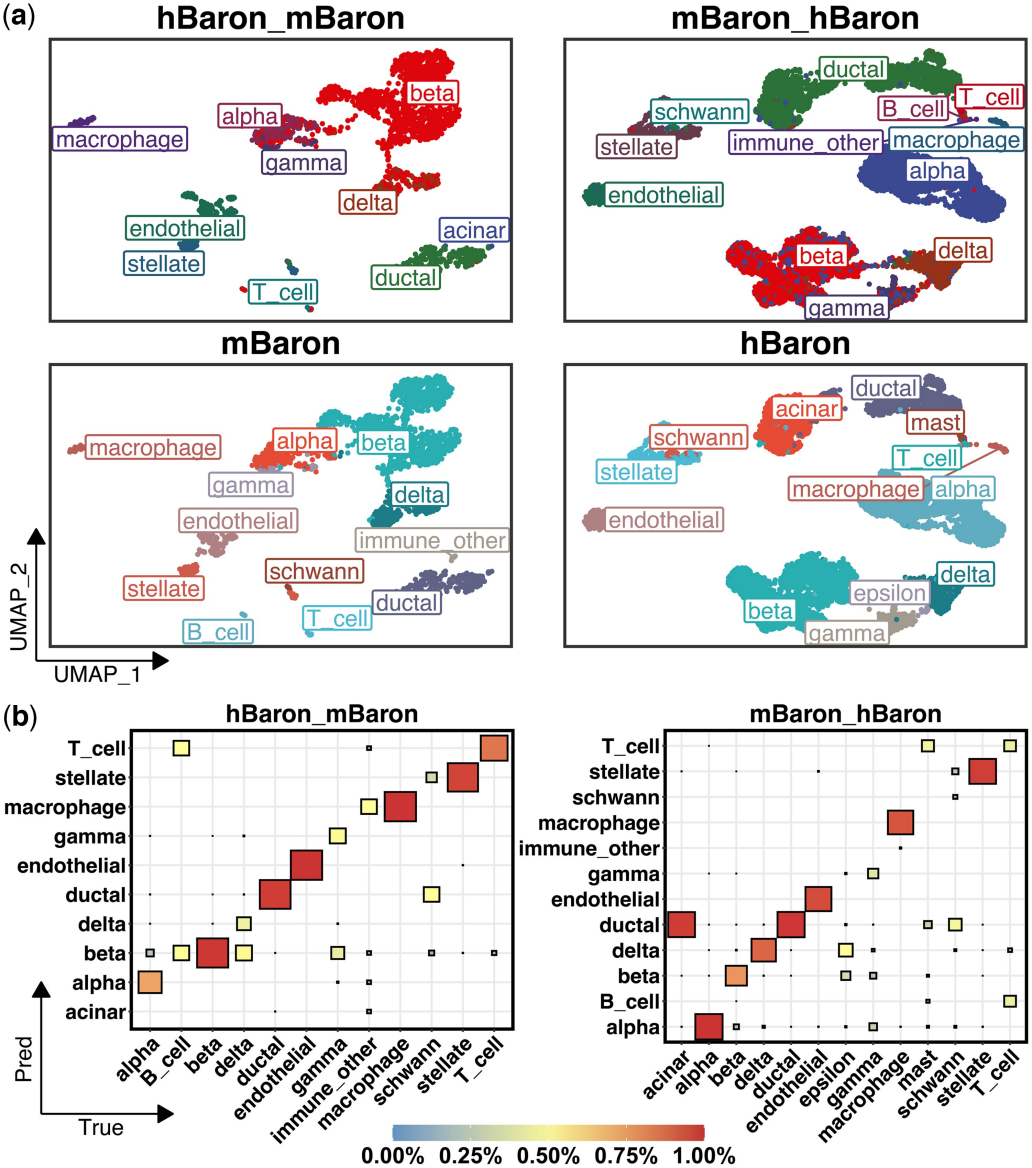
(a) The UMAP visualization of comparison of PredGCN identification results and true labels in cross-species scenarios. In each column, the top one is the identification result of PredGCN, and the bottom one is the real label of the query dataset. (b) The heatmap plot of proportion of cells in each row with true labels T (True, shown on the bottom) predicted as cell type P (Prediction, shown on the left).

To evaluate the identification ability of PredGCN in cross-species scenarios, we used the Uniform Mobility Approximation and Projection (UMAP) technique to visualize the vertical and putative labels of PredGCN in comparison with the chromatic encoding. As shown in Fig. 3a and Supplementary Fig. S5, we find that the imputed labels by PredGCN closely align with the ground truth, demonstrating high fidelity and realism. Given the potential ontological differences in cell taxonomies between the reference and query datasets, it is tenable that the cell type identification output and the genuine labels may comprise incongruous cell phenotypes. For instance, the ’acinar’ cell type, present in the hBaron dataset, is absent from the mBaron dataset in Fig. 3a. Such discrepancy also highlights the risk of misclassifying ’acinar’ phenotypes when applying parameters from the hBaron training to analyze the mBaron data, underscoring the importance of careful parameter selection. Moreover, to exhibit the superiority of PredGCN through intuitive yet rigorous manifestations, we employed heatmap visualizations to depict the consistency intervals between PredGCN’s identification results and the ground truth labels in Fig. 3b and Supplementary Fig. S6. In Supplementary Section 3, we further discuss the case presented in Fig. 3b, where acinar cells in the mBaron_hBaron dataset were usually predicted as ductal cells. These visualizations reveal PredGCN’s precision in identifying homologous cells across different datasets, maintaining high precision and recall rates. This efficiency in accurate phenotype mapping persists even when the datasets’ origins vary, demonstrating PredGCN’s robustness in cell type identification.

### 3.5 Effects of PrO on PredGCN

**Figure 4.**
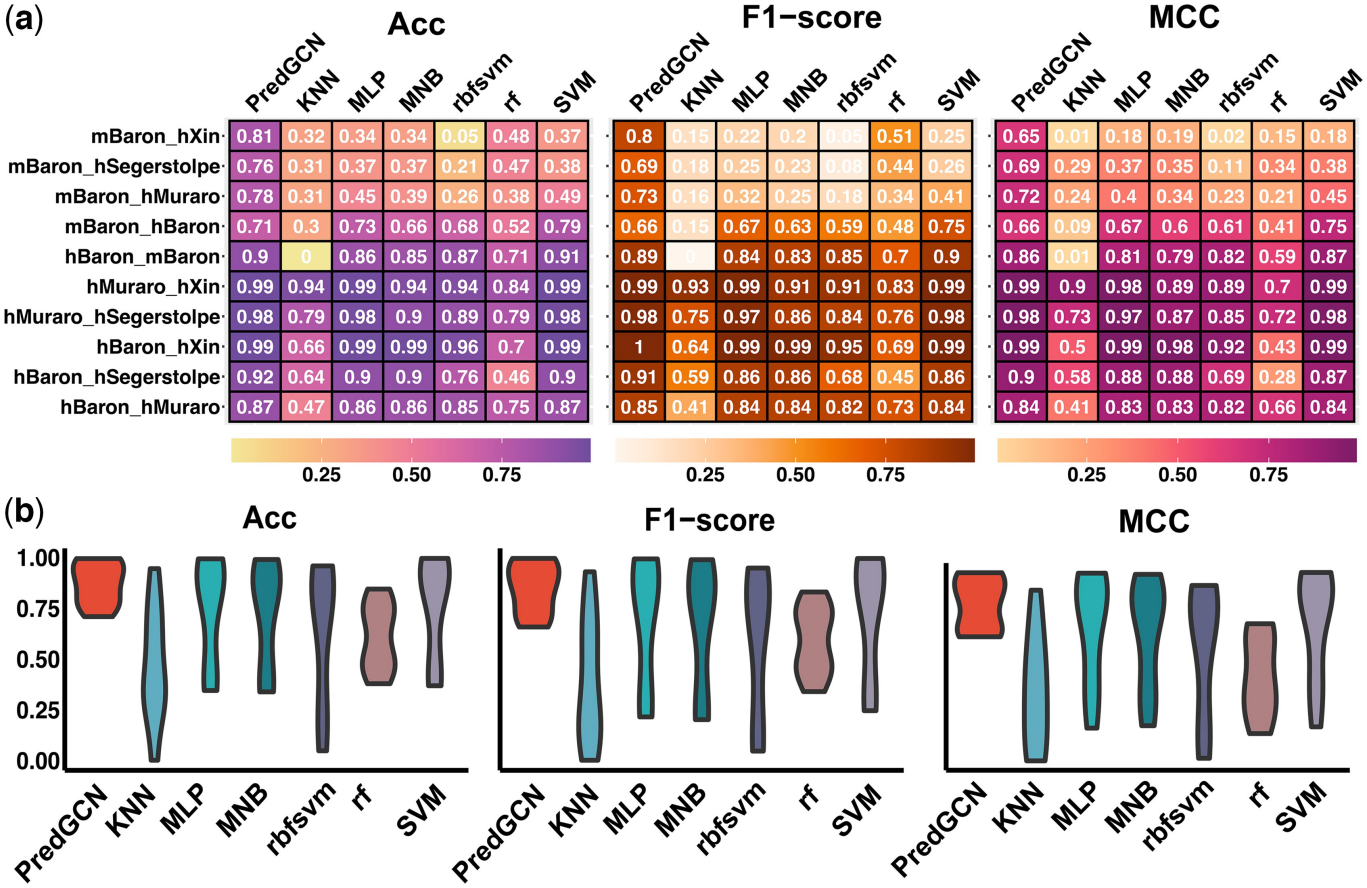
(a) The heat map of Acc, F1-score, and MCC values of PredGCN and six standalone machine learning classifiers. (b) The violin plot of Acc, F1-score, and MCC values of PredGCN and six standalone machine learning classifiers.

To validate the advanced inferential capabilities introduced by the PrO feature in the PredGCN architecture, we conducted a comprehensive benchmark analysis. This analysis compared PredGCN’s performance with that of individual machine learning classifiers integrated within PredGCN as CSN, including KNN, MLP, MNB, rbfsvm, rf, and SVM. The comparison spanned across both same-species and cross-species datasets, totaling five datasets each. As quantitatively elucidated in Fig. 4a, PredGCN outperforms standalone classifiers on 7 of 10 datasets across all three metrics. An aggregated view of the evaluation metrics, shown in Fig. 4b, reveals that PredGCN achieved average Acc, F1-score, and MCC of 0.871, 0.850, and 0.828, respectively, significantly outperforming the compared methods. When stratified by species-specific use cases, PredGCN exhibited notably higher Acc (0.953), F1-score (0.946), and MCC (0.940) compared to MLP and SVM in same-species contexts, as seen in Supplementary Fig. S8. However, in cross-species scenarios, the benefits of ensemble pruning became distinctly evident. PredGCN demonstrated marked improvements across all metrics (Acc: 0.790; F1-score: 0.753; MCC: 0.716), highlighting a significant performance degradation among standalone classifiers in such scenarios, as illustrated in Supplementary Fig. S9. This robustness in cross-species analyses is attributed to the selective retention of high-performing classifier sub-networks during the pruning process. Overall, these results underscore PredGCN’s capability to not only match but also surpass the performance of its constituent classifiers across diverse datasets, achieving enhanced robustness through pruning.

### 3.6 PredGCN can identify unknown cell types that are not present in the reference dataset

We implemented PredGCN in human pancreas data to evaluate its ability to identify novel cell types not present in the reference dataset. The reference comprised aggregated data from hMuraro, hSegerstolpe, and hXin, limited to four predominant types: alpha, beta, delta, and gamma. For the analysis, cells within the hBaron dataset were classified using PredGCN’s optimal sub-network, which included additional cell types such as acinar, ductal, and endothelial, categorized as unknown. To ascertain the impact of the rejection threshold on PredGCN’s performance, we investigated thresholds from 0.5 to 0.95 in 0.05 increments, aiming to enhance the detection of novel cell types. The analysis revealed that at a 0.65 rejection threshold, the model achieved its peak performance, with Acc, F1-score, and MCC reaching 0.886, 0.882, and 0.844, respectively (Supplementary Fig. S10). Thresholds below 0.65 resulted in the misclassification of some unknown cells as either alpha or beta cells, whereas thresholds above 0.75 led to the incorrect labeling of most delta and gamma cells, as well as some alpha and beta cells, as unknown. At the extreme threshold of 0.95, virtually all cells were classified as unknown, indicating a significant overgeneralization (Supplementary Fig. S11). Meanwhile, as shown in Supplementary Fig. S12, the performance is still highest at a rejection threshold of 0.65. In Supplementary Section 6, we further validate the robustness of the threshold 0.65 across different datasets on the human Peripheral blood mononuclear cells (PBMC) dataset (Ding *et al*. 2020). It can be observed that a rejection threshold of 0.65 can detect cell types that are different between the reference dataset and the query dataset.

### 3.7 PredGCN assists disease identification

**Figure 5.**
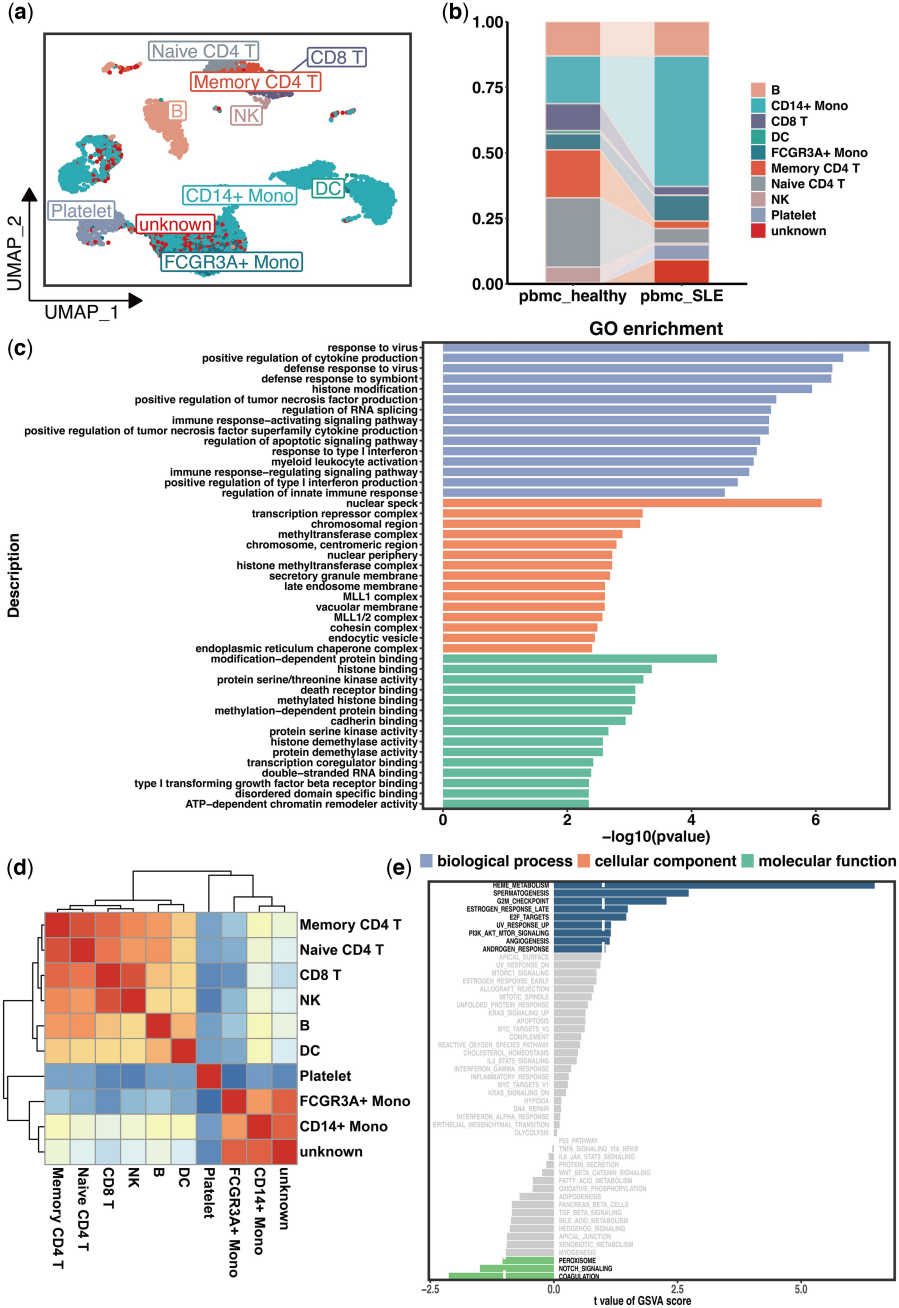
(a) 2D-Visualization of cell type identification result of SLE dataset by PredGCN via UMAP. (b) The proportion of cell types in healthy PBMC and SLE PBMC. (c) GO enrichment of unknown cells. (d) The heatmap of the correlation between cell types in the SLE dataset. (e) Differences in pathway activities scored per cell by GSVA, between unknown cells and monocytes.

To substantiate the biological relevance of single-cell identifications via PredGCN, various genomic analyses were performed. Using the PredGCN model and the healthy PBMC dataset, cellular subsets were classified within the SLE PBMC dataset. Visualization via UMAP revealed approximately 9.2% of cells as phenotypically uncharacterized, denoted as unknown (Fig. 5a, Supplementary Fig. S14), denoting the presence of populations with no prior phenotypic characterization. Comparative analysis of cell compositions between healthy and SLE PBMC datasets (Fig. 5b) highlighted significant shifts in CD4+ T cells and CD14+ monocyte levels, reflecting known SLE immunopathology and validating PredGCN’s accuracy. Further studies of the differentially expressed signature transcripts of each classified cell subpopulation are shown in Supplementary Fig. S15. To elucidate the functional properties and disease relevance of the hitherto phenotypically undefined cell subpopulations, highly expressed genes were identified on the basis of differences from unknown cell subpopulations.

The Gene Ontology (GO) enrichment analysis (Fig. 5c) highlighted an overrepresentation of immune response activation and type I interferon signaling pathways, essential in SLE’s heterogeneity and pathogenesis (Deng *et al.* 2021). Furthermore, genomic enrichment analysis indicated a downregulation of ribosome-associated pathways in the unidentified cells (Supplementary Fig. S16), mirroring decreased ribosome presence in SLE’s inflammatory cells (Zhu *et al.* 2023). These comprehensive genomic studies underscore the biological relevance of the unknown cells identified by PredGCN in SLE’s immunopathology, affirming their potential impact on disease understanding and treatment approaches.

In addition, to further explore the potential biological significance of the unknown cell types, the correlations between different cell clusters were computed. The correlation in Fig. 5d indicates that the unknown cells are highly relevant to the monocytes. Existing research elucidates that alterations in monocytes are associated with the pathogenesis of systemic lupus erythematosus (Nehar-Belaid *et al.* 2020). We speculate that potential biomarkers or therapeutic targets for SLE may be discovered by comparison between unknown cells and monocytes. We performed GSVA on unknown cells and monocytes. Figure 5e demonstrates that HEME_METABOLISM is the most enriched signature in unknown cells, suggesting that HEME_METABOLISM-related signaling in cells may be involved in SLE.

For the differential genes, we performed GO enrichment analysis to detect enriched functional attributes based on gene-related GO terms (Supplementary Fig. S17). We observe that the top enriched GO biological process is protein folding, the top enriched GO cellar component is endoplasmic reticulum chaperone complex, and the top enriched GO molecular function is peptidyl-prolyl cis-trans isomerase activity. The enrichment of these related pathways has the potential to provide guidance for the biological diagnosis of SLE and the development of therapeutic regimens. Meanwhile, we also performed KEGG pathway analysis to investigate the molecular pathways behind the enrolled genes (Supplementary Fig. S18).

Additionally, we constructed a protein–protein interaction (PPI) network of the DEGs of unknown cell types. From Supplementary Fig. S19, the top 10 hub genes identified are H4C6, HSPA5, HNRNPH1, UBB, HSP90B1, SREK1, TOP1, ZRANB2, PPIB, and PRDM2. These genes or proteins may serve as potential biomarkers for SLE, providing insights and directions for further research. In summary, the results indicate that PredGCN’s analysis of SLE can elucidate potential regulatory mechanisms and guide the identification of new therapeutic targets for SLE.

## 4 Discussion

To overcome the problem of dependency on classifier architecture in machine learning and deep learning methods for cell type identification, we introduce the PredGCN framework, designed for the automatic annotation and comprehensive characterization of single-cell transcriptomic heterogeneity. PredGCN utilizes CGCN for representation learning and information storage, taking advantage of the complementary strengths of the various classifiers to improve the robustness and accuracy of the model. A multi-objective Pareto pruning (PrO) operation is then applied to ensure that only the most contributing sub-network is retained, thus reducing architectural dependencies while maintaining high performance. Extensive comparative analyses validate PredGCN’s superiority in cell type identification and characterization, demonstrating its cutting-edge effectiveness. While PredGCN is superior, it incurs additional computational overheads and depends on the diversity and quality of the initial classifiers. In addition, the quality of the reference dataset also has an impact on the identification results. In future work, we will optimize the computational efficiency and further improve the diversity of the initial classifiers, in addition to further improving the robustness of PredGCN under reference datasets of different qualities.

## Supplementary Material

btae421_Supplementary_Data

## Data Availability

The data supporting this article are hosted on a dedicated webserver, available at http://www.aibio-lab.com/PredGCN/index/. The source code is available at https://github.com/IrisQi7/PredGCN and test data is available at https://figshare.com/articles/dataset/PredGCN/25251163.
