# Supplementary material for: PredGCN: a Pruning-enabled Gene-Cell Net for automatic cell annotation of single cell transcriptome data
Source: Bioinformatics. 2024 Jun 26;40(7):btae421. doi: 10.1093/bioinformatics/btae421 (PMC11236098; doi:10.1093/bioinformatics/btae421)
Supplement: btae421_Supplementary_Data [file btae421_supplementary_data.pdf]

Supplementary Material to "PredGCN: A Pruning-enabled Gene-Cell Net for Automatic Annotation of Single Cell Transcriptome Data"

Contents

1 Section 1: Results for 3.3 2

2 Section 2: Results for 3.4 3

3 Section 3: Misclassification of Acinar Cells in PredGCN 5

4 Section 4: Results for 3.5 6

5 Section 5: Results for 3.6 7

6 Section 6: Robustness and Adaptability of PredGCN Thresholds in Identifying Cell Types Across Diverse Datasets 9

7 Section 7: Results for 3.7 11

8 Section 8: Details about gene filtering sub-nets 15

9 Section 9: Evaluation metrics 16

10 Section 10: Hyperparameter selection discussion for competitors 16

11 Section 11: Supplement to PredGCN Webserver 16

12 Section 12: Summary of Datasets 17

13 Section 13: Tutorial of PredGCN Webserver 18

1 Section 1: Results for 3.3

Figure S1, S2, S3, S4.

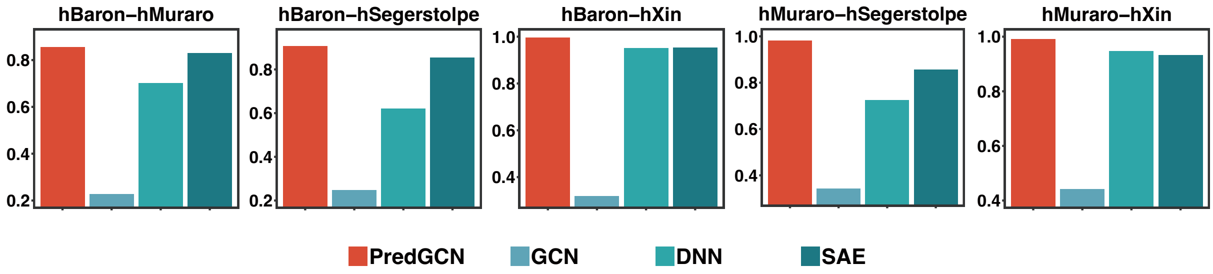

Figure S1: Comparison of F1-score between PredGCN and 3 deep learning methods in same-species scenarios. The F1-score of PredGCN ranked first on all datasets.

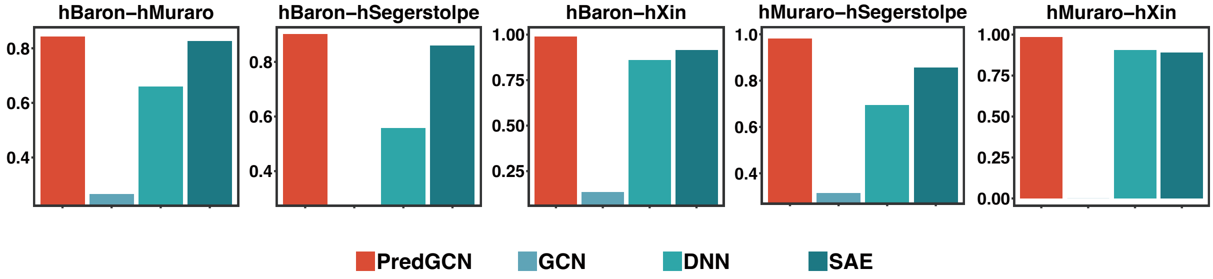

Figure S2: Comparison of the MCC of PredGCN with 3 deep learning methods in same-species scenarios. The MCC of PredGCN outperforms the comparison algorithms on all datasets.

	F1-score					
Method	hBaron-hMuraro	hBaron-hSegerstolpe	hBaron-hXin	hMuraro_hSegerstolpe	hMuraro_hXin	Median
PredGCN	0.8547	0.9068	0.9953	0.9816	0.9922	0.9816
SingleCellNet	0.8803	0.9137	0.9912	0.9781	0.9905	0.9781
SingleR	0.9457	0.9572	0.9671	0.984	0.9883	0.9671
CaSTLe	0.7961	0.6689	0.8866	0.9033	0.9529	0.8866
scmap−cluster	0.8322	0.7388	0.9245	0.9518	0.9409	0.9245
scmap−cell	0.74	0.7038	0.8963	0.8739	0.9453	0.8739

Figure S3: Comparison of F1-score of PredGCN with 5 representative algorithms. The results of PredGCN are ranked ahead of all algorithms and the median F1-score of PredGCN is ranked first.

	MCC					
Method	hBaron-hMuraro	hBaron-hSegerstolpe	hBaron-hXin	hMuraro_hSegerstolpe	hMuraro_hXin	Median
PredGCN	0.8429	0.902	0.9889	0.9801	0.9852	0.9801
SingleCellNet	0.858	0.9056	0.984	0.9758	0.9827	0.9758
SingleR	0.9246	0.9477	0.9327	0.9826	0.979	0.9477
CaSTLe	0.7693	0.5801	0.8041	0.8812	0.9104	0.8041
scmap−cluster	0.7701	0.7012	0.8413	0.8996	0.8271	0.8271
scmap−cell	0.6875	0.6726	0.8154	0.8164	0.8644	0.8154

Figure S4: The comparison of MCC of PredGCN with 5 representative algorithms. The MCC of PredGCN is competitive with the other algorithms and the median MCC of PredGCN is ranked on top.

2 Section 2: Results for 3.4

Figure S5, S6

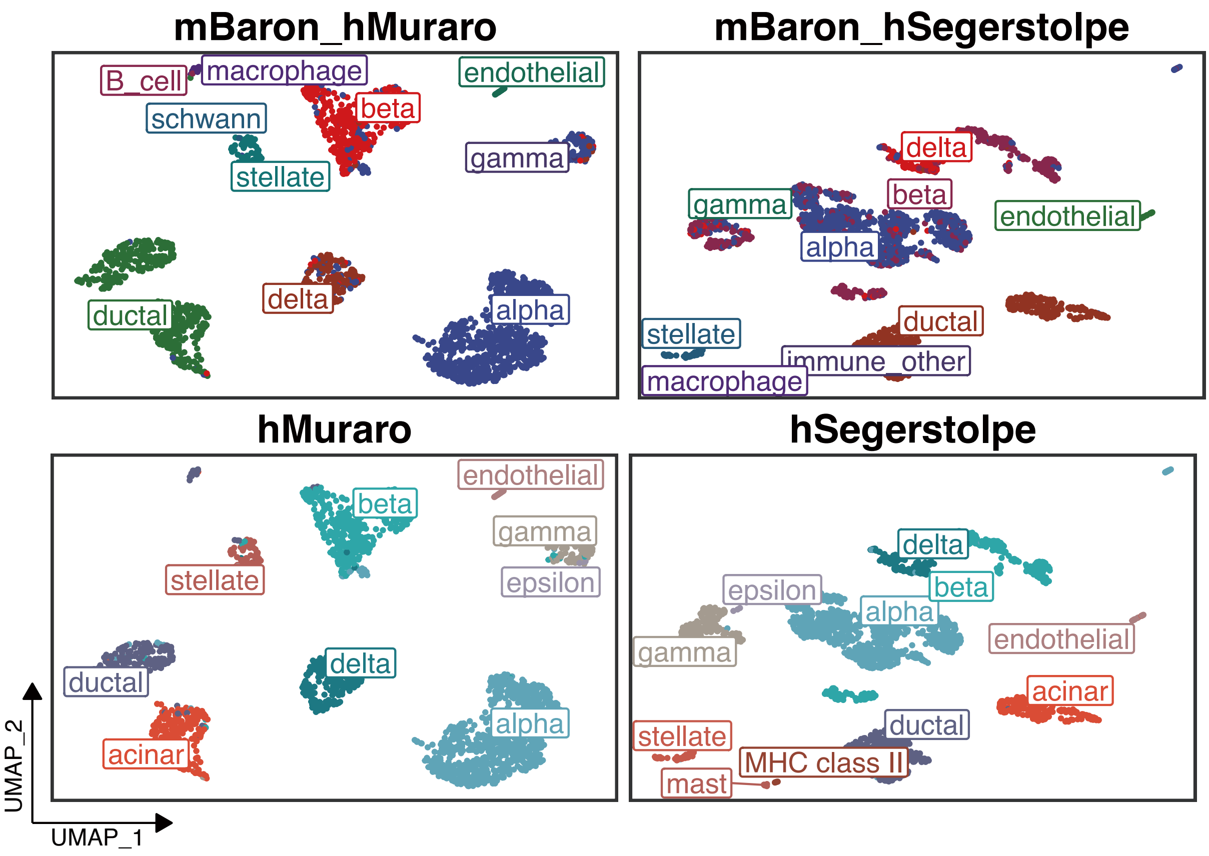

Figure S5: UMAP visualization of comparison of PredGCN identification results and true labels in cross-species scenarios. In each column, the top one is the identification result of PredGCN, and the bottom one is the real label of the query dataset.

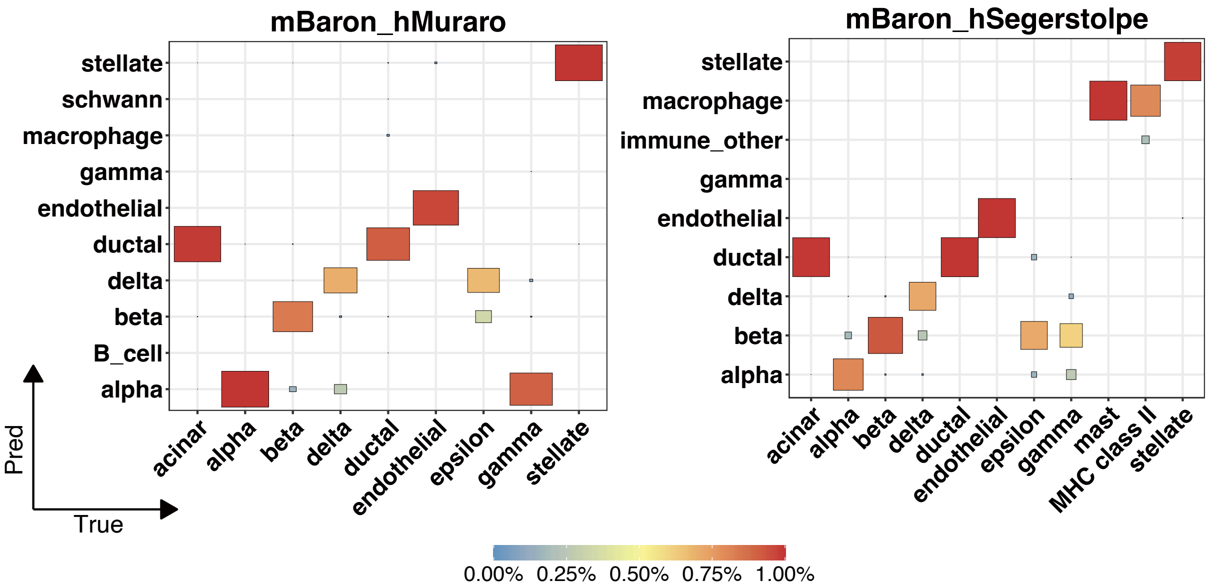

Figure S6: Heatmap shows proportion of cells in each row with true labels T (True, shown on the bottom) predicted as cell type P (Prediction, shown on the left).

3 Section 3: Misclassification of Acinar Cells in PredGCN

In Fig. 3b, we observe that acinar cells in the mBaron_hBaron dataset are predicted as ductal cells. This misclassification occurs because acinar cells are absent in the mBaron dataset, which serves as the reference dataset. Consequently, the model trained on the mBaron dataset lacks data on acinar cells, resulting in an inability to establish effective classification criteria and features for these cells, leading to limitations and inaccuracies in their identification.

The primary reason acinar cells are often misidentified as ductal cells is due to their close relationship within the exocrine pancreas. Acinar and ductal cells are closely related in both location and function (Zhou and Melton, 2018). As indicated in Figure S7, the correlation between acinar and ductal cells is more pronounced than with other cell types. Therefore, when the model lacks information on acinar cells, it may misidentify these cells in the hBaron dataset as ductal cells.

We acknowledge that PredGCN currently has some limitations. PredGCN relies on a reference dataset for cell annotation, necessitating high quality and comprehensiveness of the reference data. If the reference dataset is incomplete or contains biases, the annotation results can be significantly impacted. This limitation affects the generalization ability of PredGCN, particularly when encountering new cell types or states.

To address these challenges, we recommend using a more comprehensive reference dataset when employing PredGCN. Avoiding the use of subset datasets for training will help improve the accuracy and generalization capability of the model, ensuring more reliable cell type identification across diverse datasets.

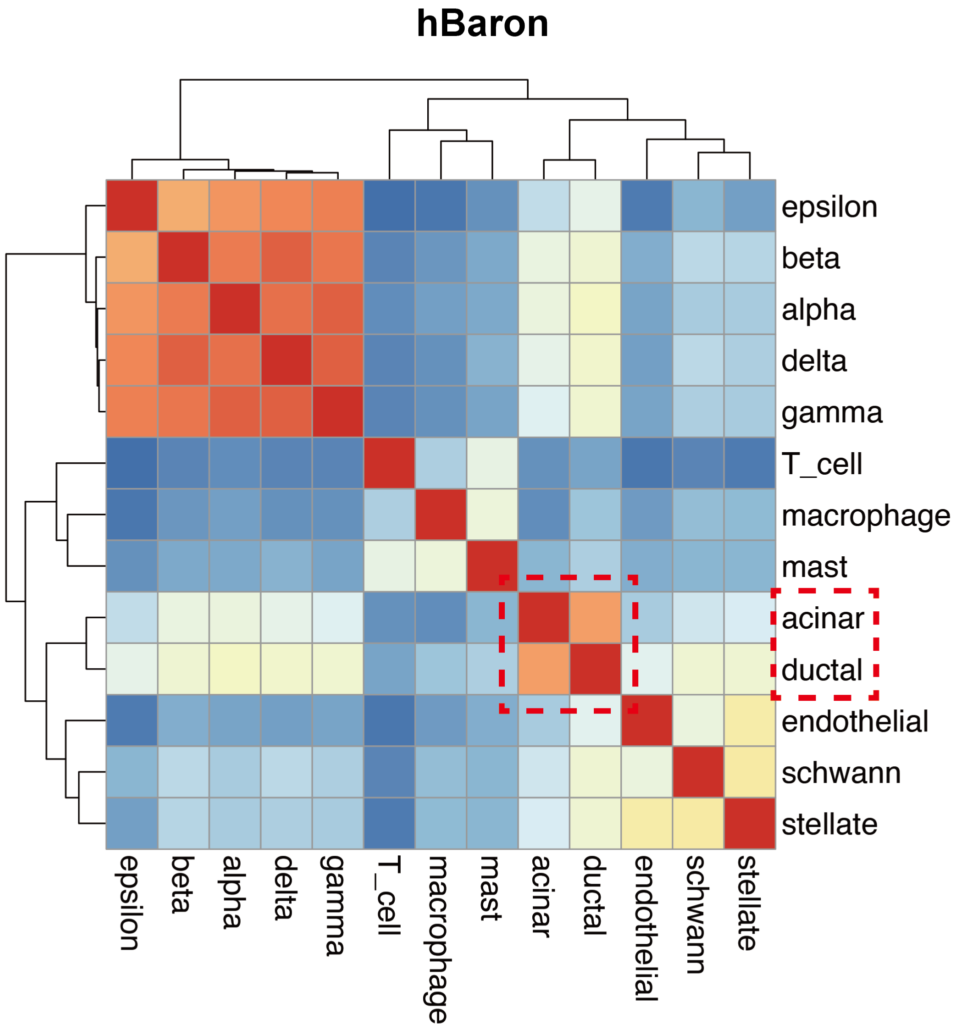

Figure S7: Correlations between cell types in the hBaron dataset. Acinar cells correlate more highly with ductal cells than with other cell types.

4 Section 4: Results for 3.5

Figure S8, S9

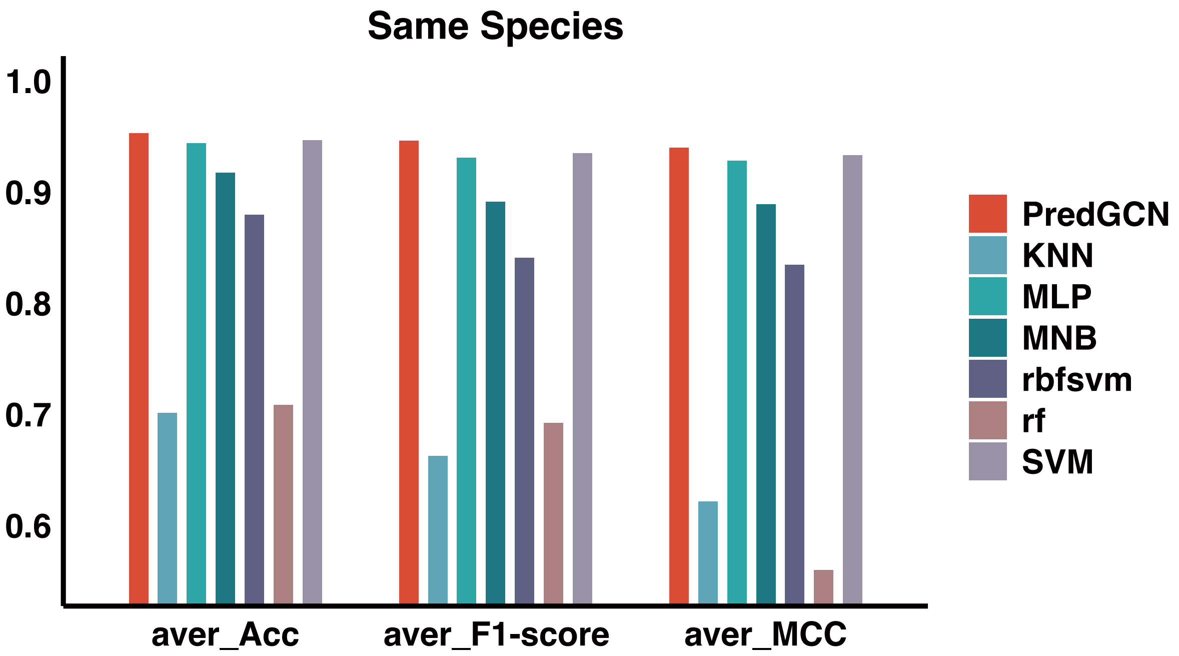

Figure S8: Comparison of average Acc, F1-score and MCC of PredGCN and compared machine learning algorithms in same-species scenarios. PredGCN ranks at the top of all metrics.

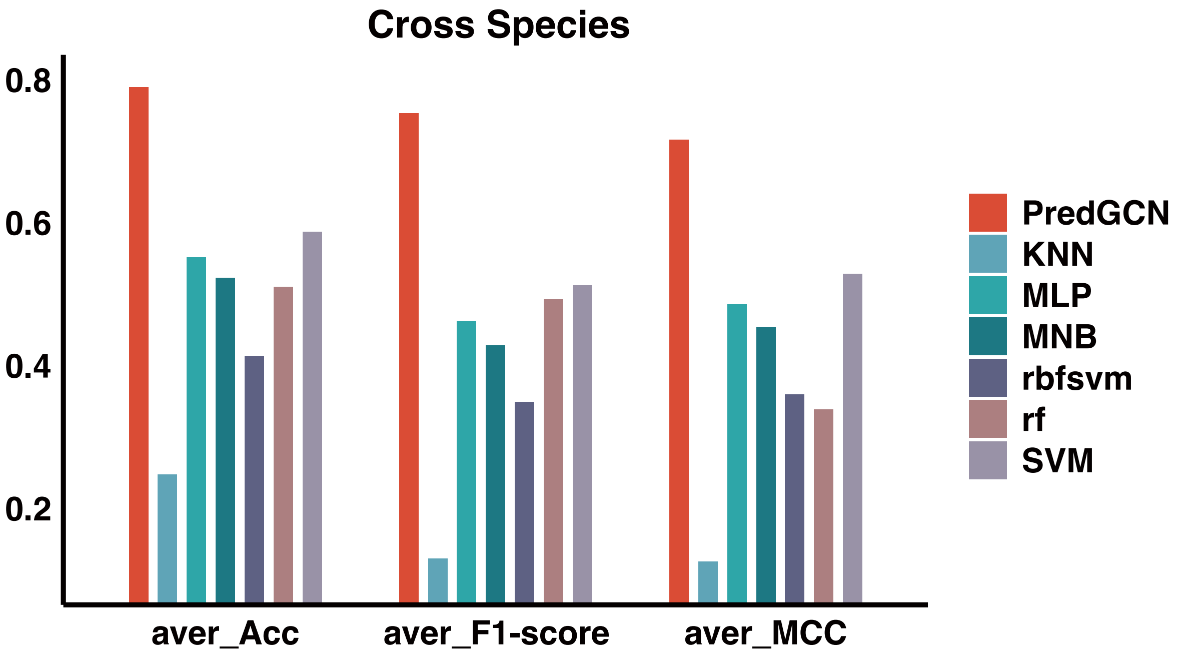

Figure S9: Comparison of the average Acc, F1-score, and MCC of PredGCN and compared machine learning algorithms in a cross-species scenario. PredGCN performs best in all metrics.

5 Section 5: Results for 3.6

Figure S10, S11, S12

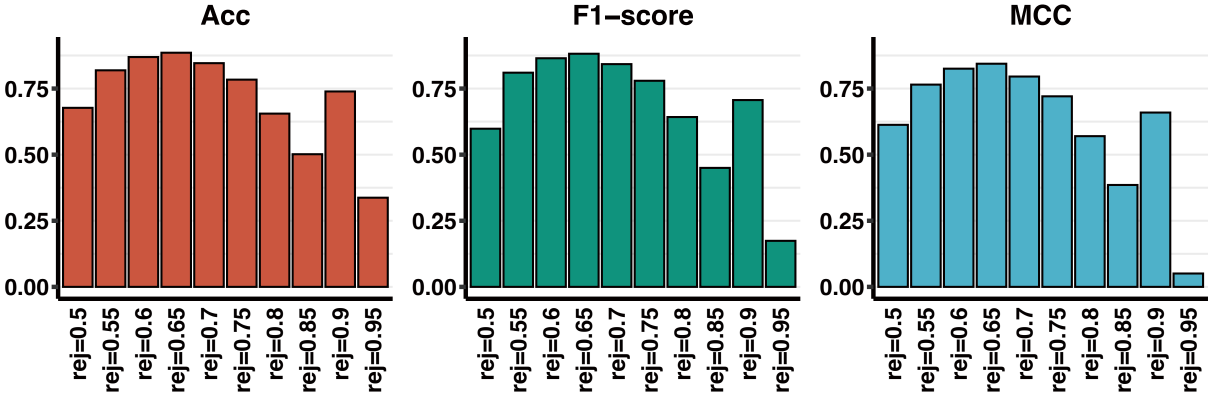

Figure S10: Comparison performance of different rejection thresholds of PredGCN measured by Acc, F1-score and MCC.

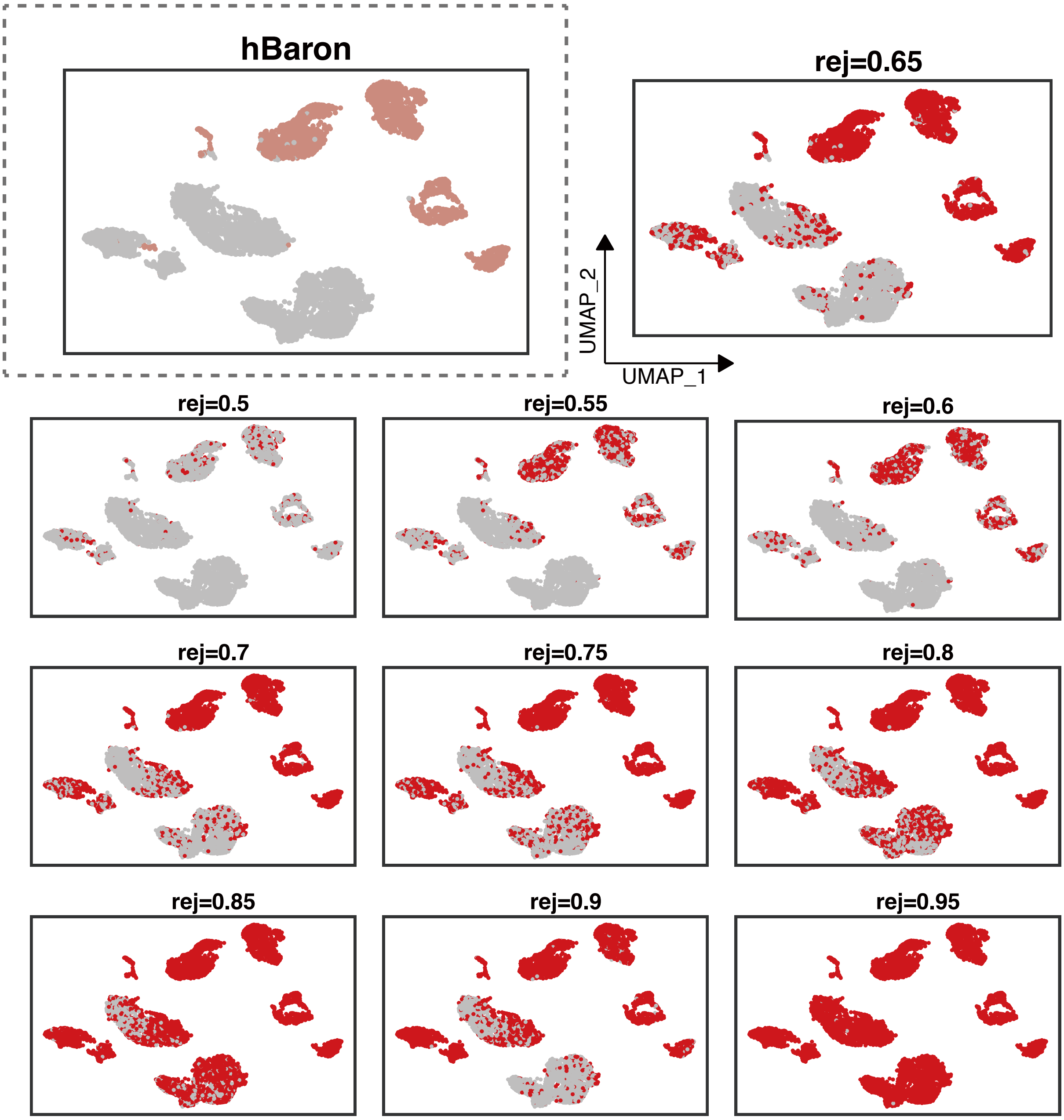

Figure S11: 2D-visualization of PredGCN performance on the hBaron dataset using UMAP for rejection threshold at 0.5, 0.55, 0.6, 0.65, 0.7, 0.75, 0.8, 0.85, 0.9, and 0.95. The reference dataset is the integration of the hMuraro, hSegerstolpe and hXin datasets.

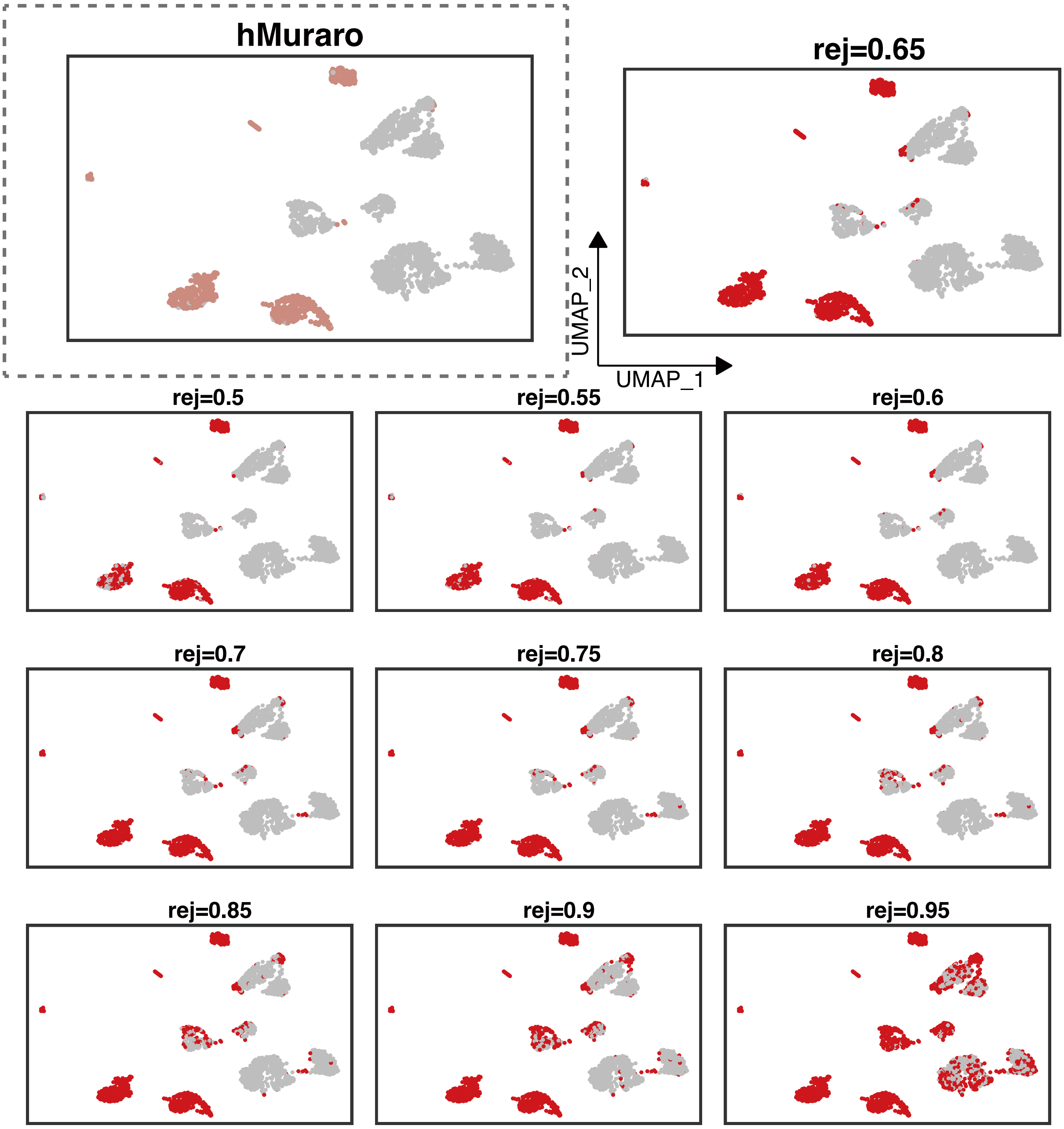

Figure S12: 2D-visualization of PredGCN performance on the hMuraro dataset using UMAP for rejection threshold at 0.5, 0.55, 0.6, 0.65, 0.7, 0.75, 0.8, 0.85, 0.9, and 0.95. The reference dataset is the hXin dataset.

6 Section 6: Robustness and Adaptability of PredGCN Thresholds in Identifying Cell Types Across Diverse Datasets

Figure S13

To evaluate the robustness of our threshold settings, we conducted validation experiments on various datasets derived from human Peripheral Blood Mononuclear Cells (PBMC) (Ding *et al.*, 2019). Figure S13 illustrates the precision of PredGCN in identifying cell types across different thresholds applied to these datasets. The thresholds tested were 0, 0.4, 0.6, 0.65, 0.7, 0.8, and 1. As shown in Figure S13(a), while a threshold of 0.65 was not the top performer across all datasets, it consistently ranked among the top three in multiple datasets. In contrast, thresholds such as 1.0 achieved higher precision in specific datasets but lacked consistent performance overall. The 0.65 threshold demonstrated balanced perfor-

mance, maintaining high precision under various data conditions.

Figure S13(b) highlights that the median precision is highest and the data distribution is more concentrated at a threshold of 0.65, indicating stable performance of PredGCN at this threshold. This stability is crucial for identifying unknown cell types in both human pancreas datasets and human PBMC datasets. However, it is important to note that the optimal threshold may vary depending on the dataset, and slight adjustments might be necessary to achieve the best results in different contexts.

Future work will involve extending threshold validation to a wider range of datasets, including those from different species and experimental conditions. Additionally, we are exploring adaptive mechanisms to dynamically optimize thresholds based on the specific characteristics of each dataset, aiming to enhance the adaptability and precision of PredGCN.

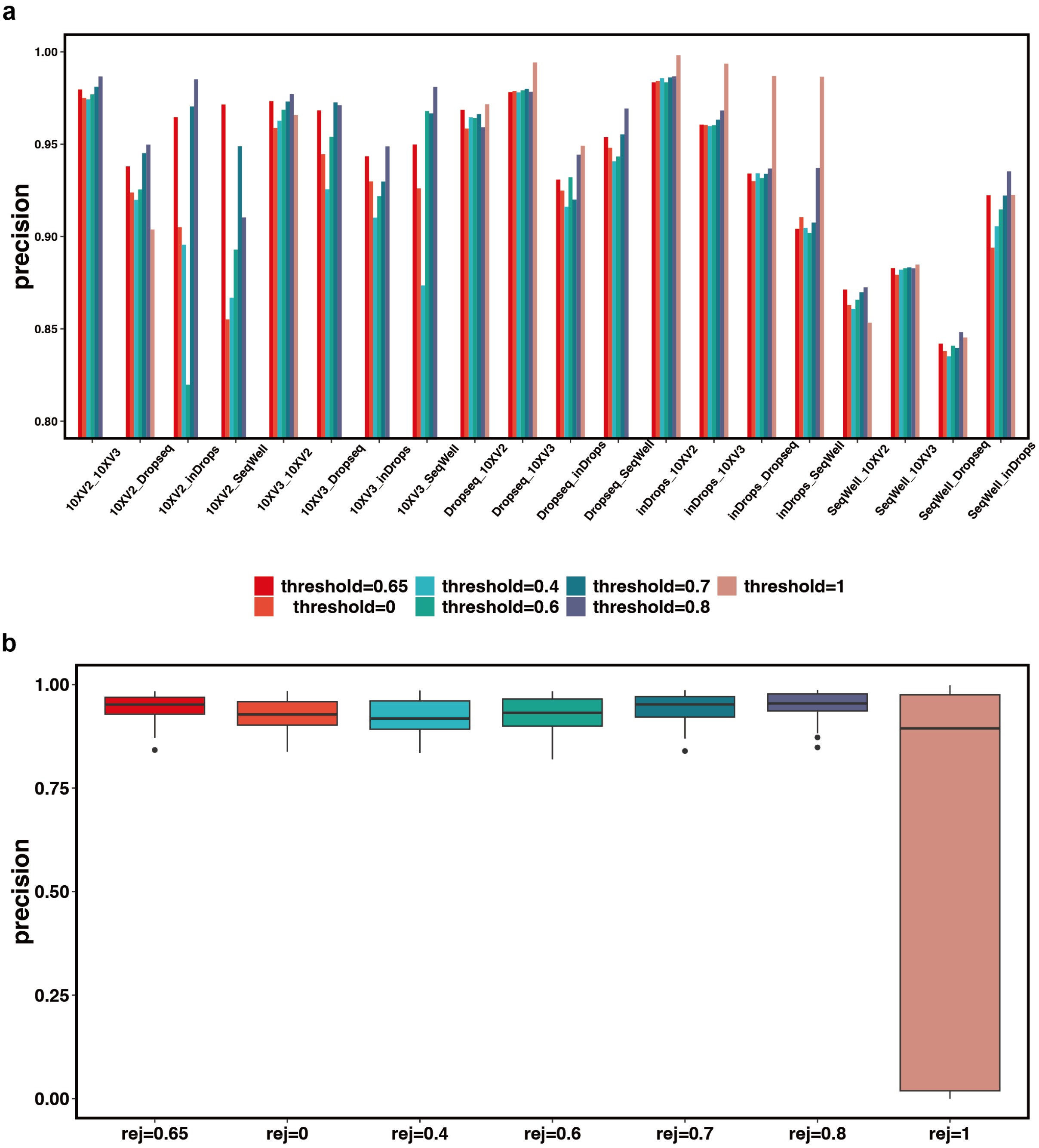

Figure S13: The performance of threshold 0.65 on PBMC datasets. (a) Comparison of the precision of PredGCN when the threshold is 0.65 and when there is no threshold. (b) The box plot shows the distribution of PredGCN precision at different thresholds (0, 0.4, 0.6, 0.65, 0.7, 0.8, and 1).

7 Section 7: Results for 3.7

Figure S14, S15, S16, S17, S18, S19

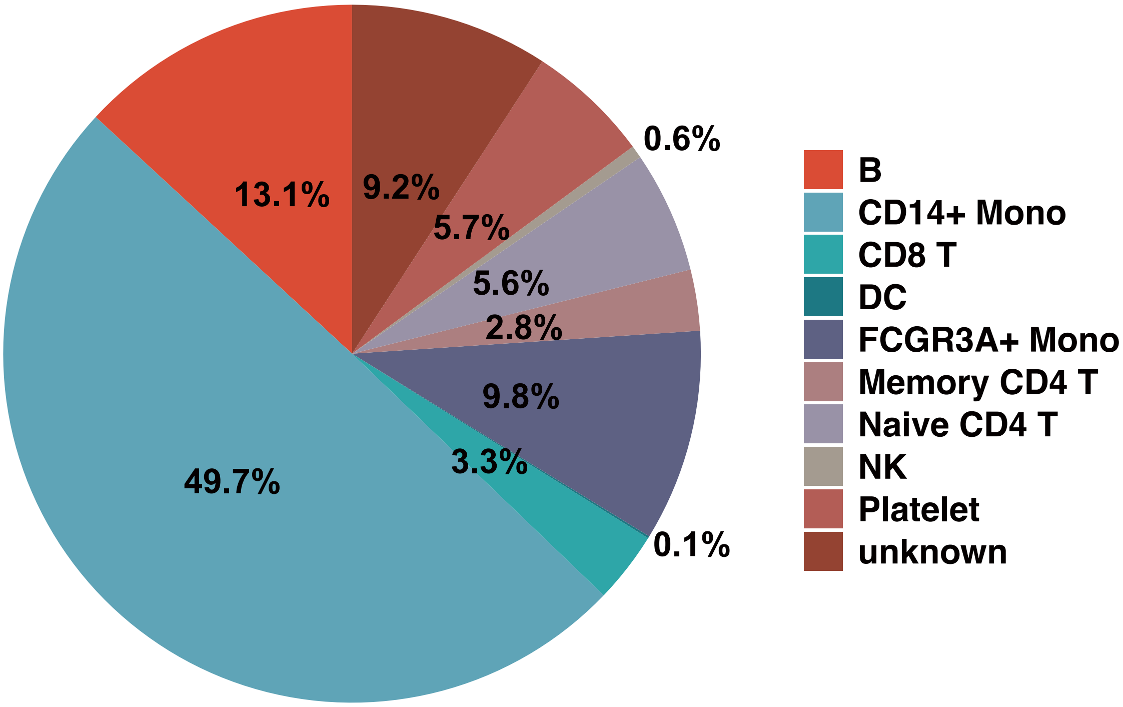

Figure S14: Pie chart of the proportion of each cell type in the results identified using PredGCN in the SLE pbmc dataset.

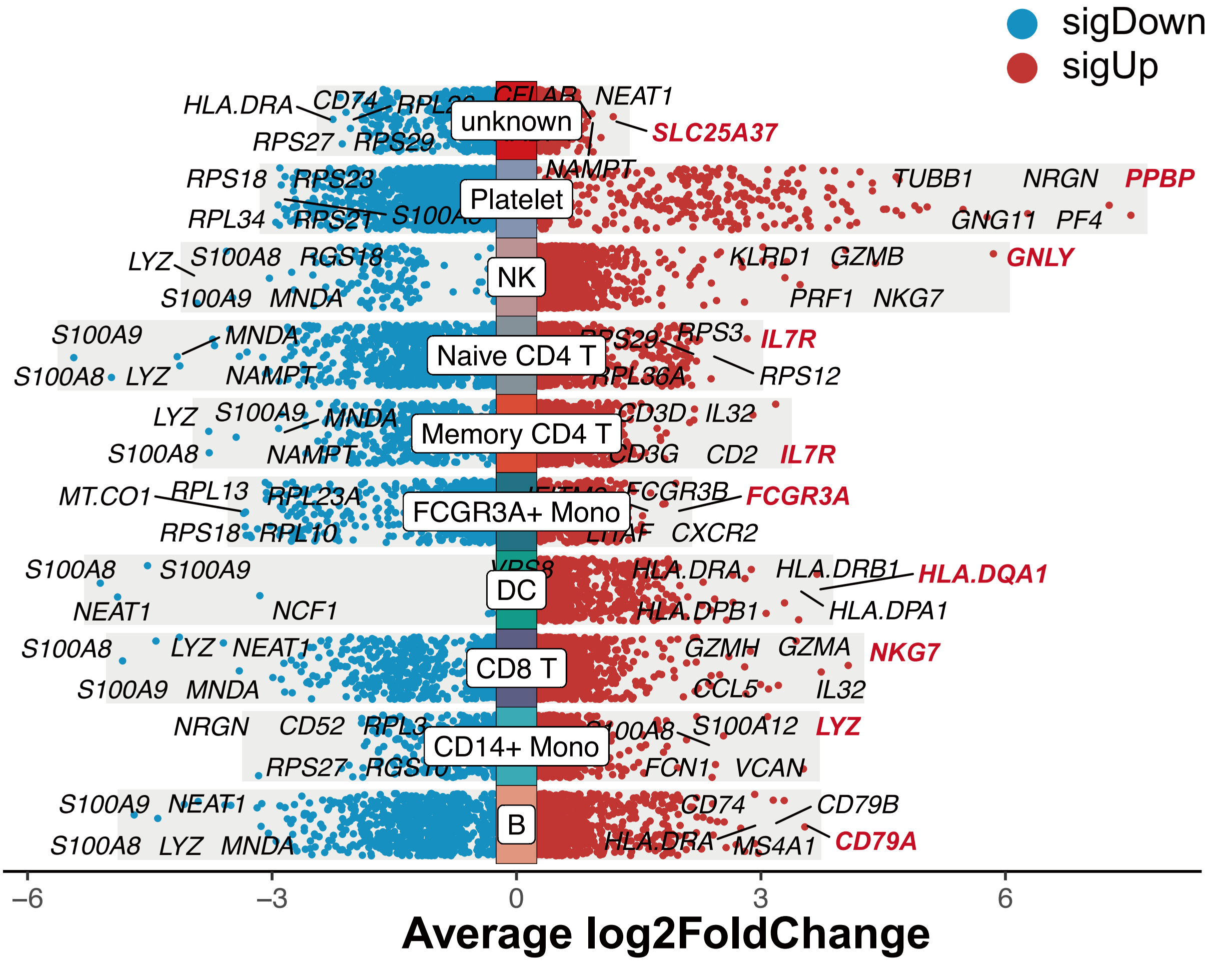

Figure S15: Visualization of expression distribution of the top marker gene of each cell type.

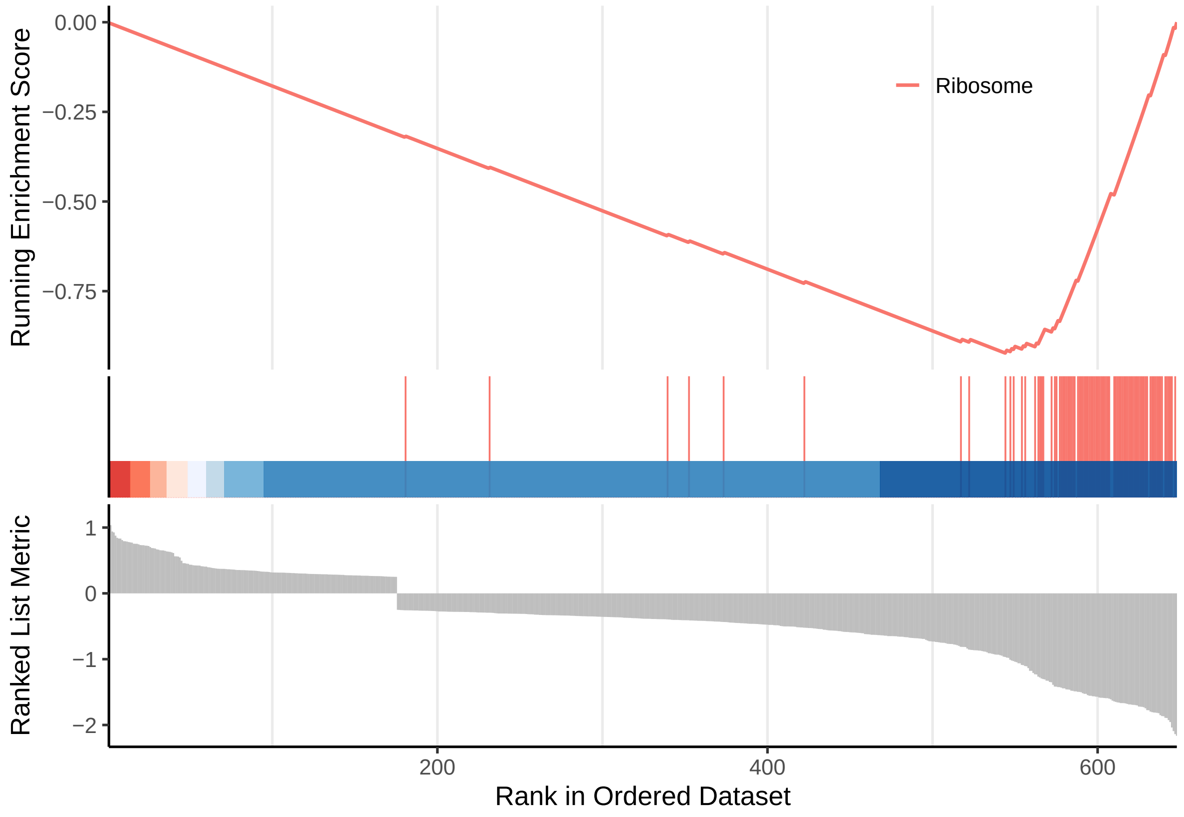

Figure S16: GSEA on KEGG pathways for unknown cells.

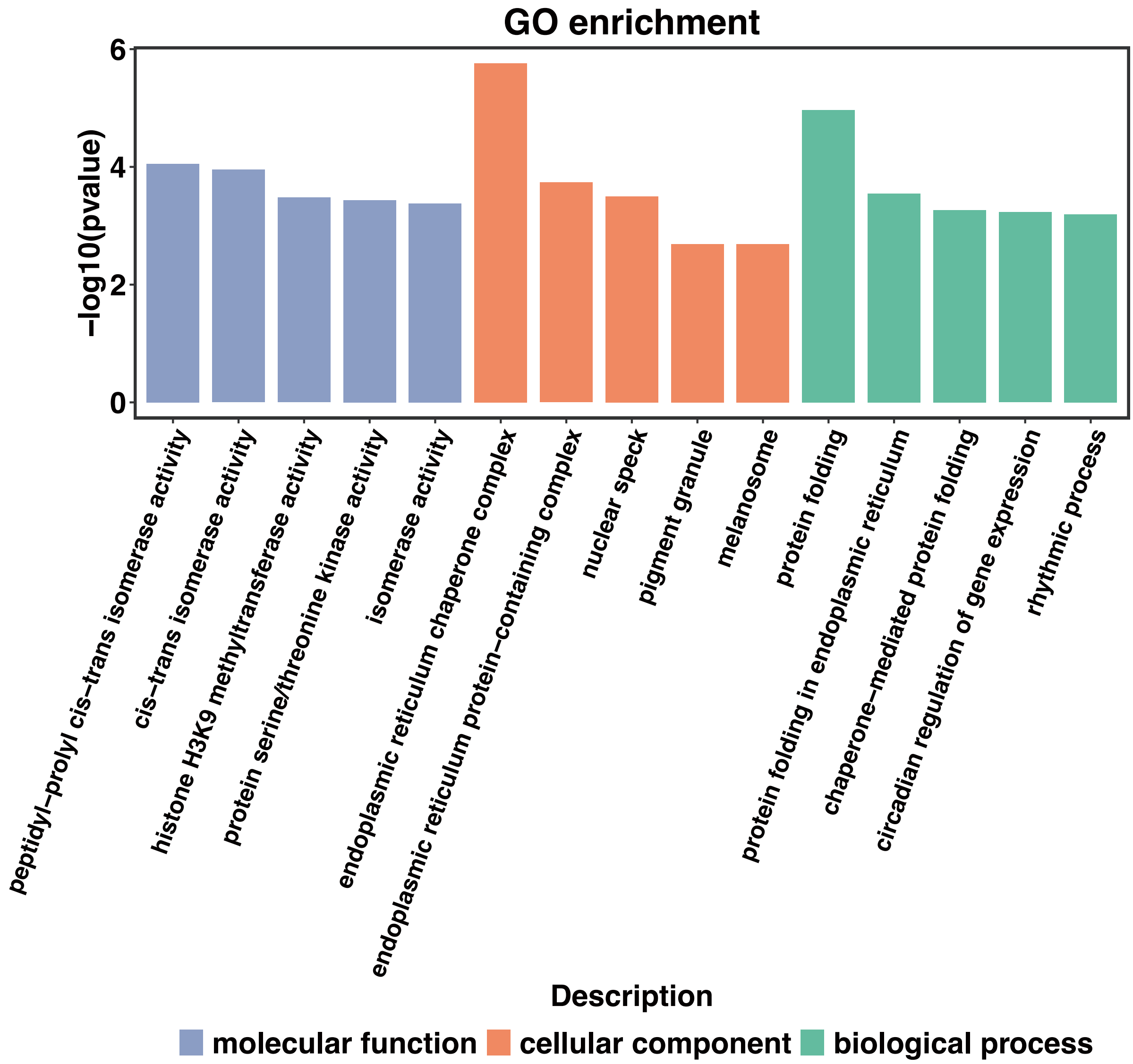

Figure S17: The distribution of gene-related GO under gene ontology (GO) enrichment for Biological Processe(BP), Cellular Component (CC), and Molecular Function in the subset of monocytes and unknown cells.

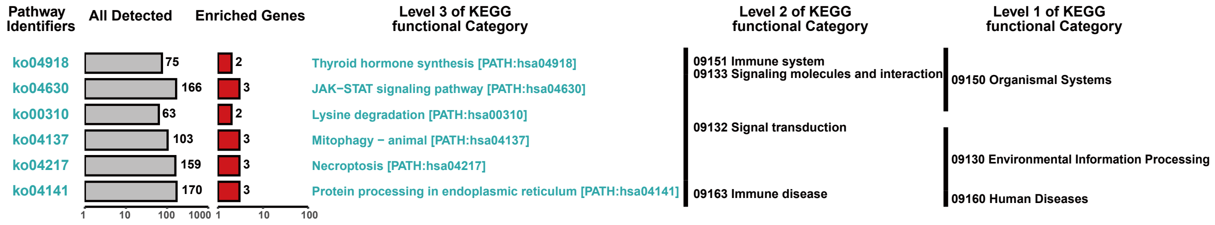

Figure S18: Three levels of KEGG functional categories and KEGG pathways of unknown cells in the subset of monocytes and unknown cells.

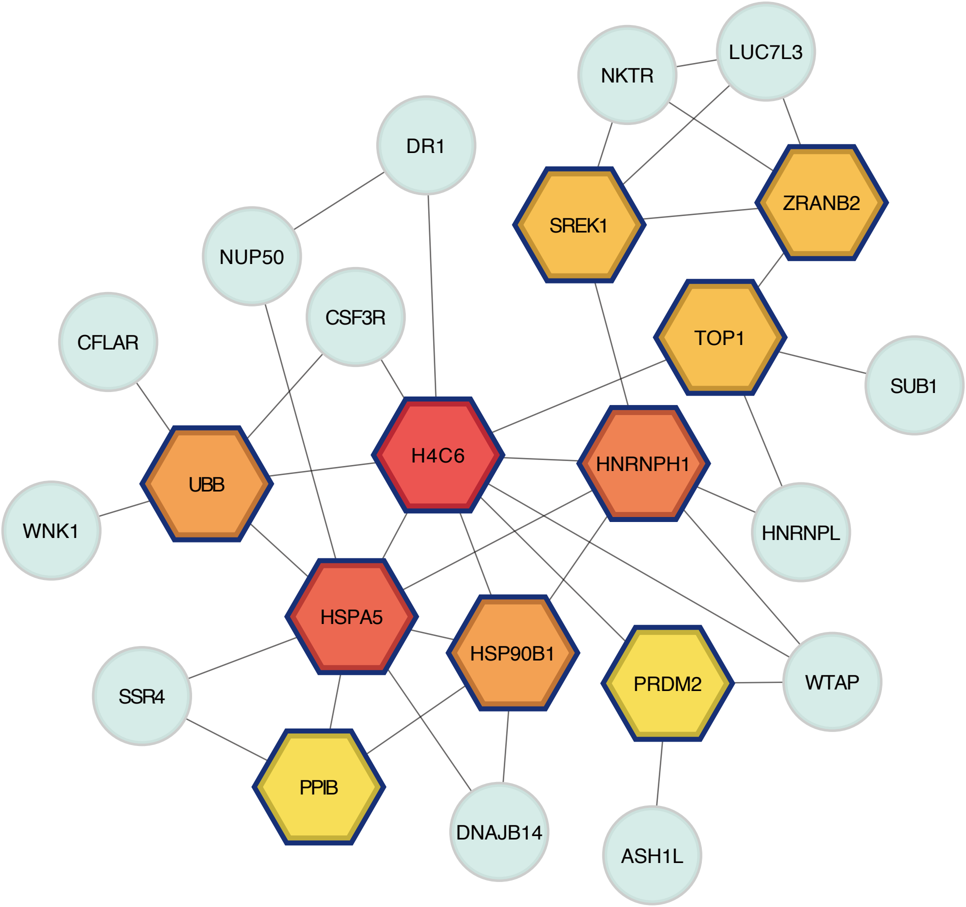

Figure S19: Collection of hub genes from the PPI network using the Cytohubba. The hexagonal-nodes indicate the top-10 hub genes. The PPI network was generated using the STRING database (https://string-db.org/) (version 12.0)(Szklarczyk *et al.*, 2023). In the network, the confidence value was set to 0.7 and the disconnected nodes were hidden. Then, the processed PPI network was put into Cytoscape (v.3.10.1) to display visually the node-to-node connections. Accordingly, Maximal Clique Centrality of cytoHubba (https://apps.cytoscape.org/apps/cytohubba) in the Cytoscape APP Store was applied to recognize the top 10 hub genes from the PPI network.

8 Section 8: Details about gene filtering sub-nets

The Bartlett's test can distinguish between differentially variant genes by homogeneity tests. The Bartlett's test is implemented using the R function bartlett.test. The detailed representations of Equation(1) are as follows:

$$W = \frac{\overline{m}}{\prod s_i^2} \tag{S1}$$

where s_i^2 is the variance of the feature i.

$$df = \frac{2}{3}(\overline{m} - 1)(\sum_{i=1}^{\overline{m}} \frac{1}{M_i - 1} - \frac{1}{\overline{n} - \overline{m}}) \tag{S2}$$

The KS test is implemented using the R function ks.test and the genes are then ranked and selected based on the p-value of the result.
For the chi-square test, genes with differential proportions are generated using the R function chisq.test (Ma *et al.*, 2020). The genes are then ranked and selected based on the p-value of the result.
For Bimodality Index, the genes are also ranked based on the T.
The detailed representations of Equation(5) are as follows:

$$df_1 = k - 1 \tag{S3}$$

$$df_2 = n_x - k \tag{S4}$$

where n_x is the total number of cells in the cell type x.

$$varb_g = \sum_{i=1}^{k} n_i \times (\overline{M^g} - \overline{M_i^g})^2 \tag{S5}$$

$$vart_g = \sum_{j=1}^{n_x} (\overline{M^g} - M_j^g)^2 \cdot \overline{M^g} \tag{S6}$$

The expression M_i^g corresponds to the expression value of the gene g in the cell i, while $\overline{M_i^g}$ and n_i denote the mean and sample size for cell type i, respectively.

9 Section 9: Evaluation metrics

Acc is mathematically defined as:

$$Acc = \frac{TP}{TP + FP + TN + FN} \tag{S7}$$

The specific derivation process for the F1-score is as follows:

$$F1 = \frac{2 \times Precision \times Recall}{Precision + Recall} \tag{S8}$$

$$Precision = \frac{TP}{TP + FP} \tag{S9}$$

$$Recall = \frac{TP}{TP + FN} \tag{S10}$$

$$\tag{S11}$$

MCC is computed from the confusion matrix as:

$$MCC = \frac{TP \cdot TN - FP \cdot FN}{\sqrt{(TP + FP)(TP + FN)(TN + FP)(TN + FN)}} \tag{S12}$$

10 Section 10: Hyperparameter selection discussion for competitors

For the DNN framework, we used two dense layers, each having 317 filters and 'relu' activation to increase the nonlinear effect. An additional Dense layer with 'sigmoid' activation followed to identify the classification result, and dropout and batch normalization were applied in the DNN to improve the generalizability of the model.

The SAE architecture had four Dense layers, with different filters from layer to layer. Here, we set the filters to 317, 159, 106 and 79 to perform a dimension reduction operation. Each layer was also followed by 'relu' activation, and an additional Dense layer with 'sigmoid' activation was added to identify the class.

For the GCN architecture, we first used KNN to obtain the adjacent relationship. The implementation of DNN and SAE used the TensorFlow and Keras libraries. The implementation of GCN used the Pytorch library. The Adam optimizer was used in the deep networks.

All the machine learning methods were run using the scikit-learn package in Python (Pedregosa *et al.*, 2011). The *alpha* of MNB was set to 0.01. The parameters of rbfsvm were set to $gamma =' auto'$ and $probability = True$. The parameter settings for rf included $n\_estimators = 10$. The max_iter of MLP was set to 500. The SVM and KNN used the default parameter settings.

11 Section 11: Supplement to PredGCN Webserver

Figure S20
Users can access our webserver via `http://www.aibio-lab.com/PredGCN/index/`.

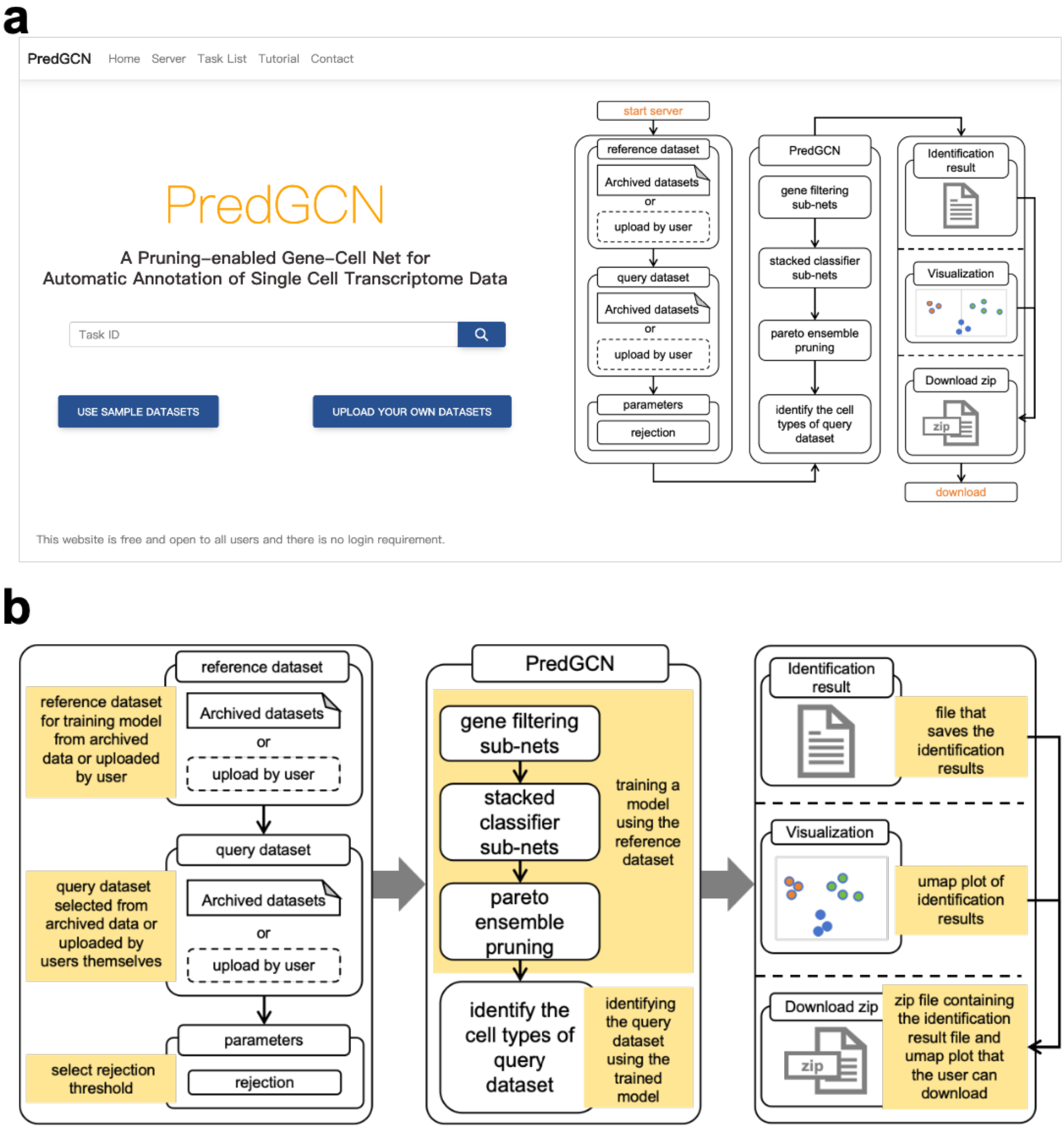

Figure S20: The (a) Home page and (b) graphical abstract of PredGCN webserver.

12 Section 12: Summary of Datasets

Table S1

Table S1: Datasets used in the manuscript.

Dataset name	Species	Protocol	Cell	Gene	Cell type	access	Detail
Muraro	Human	CEL-seq2	2212	18353	9	GSE85241	acinar:219 alpha:812 beta:448 delta:193 ductal:245 endothelial:21 epsilon:3 gamma:101 stellate:80
Segerstolpe	Human	Smart-seq2	2127	18353	11	E-MTAB-5061	acinar:185 alpha:886 beta:270 delta:114 ductal:386 endothelial:16 epsilon:7 gamma:197 mast:7 MHC class II:5 stellate:54
Xin	Human	SMARTer	1492	18353	4	GSE81608	alpha:886 beta:472 delta:49 gamma:85
Baron	Human	inDrop	8569	12474	13	GSE84133	acinar:958 alpha:2326 beta:2525 delta:601 ductal:1077 endothelial:252 epsilon:18 gamma:255 macrophage:55 mast:25 schwann:13 stellate:457 T_cell:7
Baron	Mouse	inDrop	1886	12474	12	GSE84133	alpha:191 B_cell:10 beta:894 delta:218 ductal:275 endothelial:139 gamma:41 immune_other:8 macrophage:36 schwann:6 stellate:61 T_cell:7
PBMC3k	Human	10x Genomics	2638	11833	9	SeuratData package (Stoeckius *et al.*, 2017)	B:344 CD14+Mono:480 CD8 T:271 DC:32 FCGR3A+Mono:162 Memory CD4 T:483 Naive CD4 T:697 NK:155 Platelet:14
pbmcsca	Human	10x Chromium (v2)	3362	33694	7	SeuratData package	B cell:862 Dendritic cell:76 Megakaryocyte:32 monocyte:486 Natural killer cell:219 Plasmacytoid dendritic cell:30 T cell:1657
pbmcsca	Human	10x Chromium (v3)	3222	33694	6	SeuratData package	B cell:346 Dendritic cell:38 Megakaryocyte:270 monocyte:452 Natural killer cell:194 T cell:1922
pbmcsca	Human	Drop-seq	6584	33694	7	SeuratData package	B cell:1405 Dendritic cell:46 Megakaryocyte:27 monocyte:555 Natural killer cell:484 Plasmacytoid dendritic cell:28 T cell:4039
pbmcsca	Human	inDrops	6584	33694	7	SeuratData package	B cell:1045 Dendritic cell:148 Megakaryocyte:145 monocyte:2282 Natural killer cell:147 Plasmacytoid dendritic cell:42 T cell:2775
pbmcsca	Human	Seq-Well	3727	33694	6	SeuratData package	B cell:527 Dendritic cell:37 Megakaryocyte:38 monocyte:1255 Plasmacytoid dendritic cell:26 T cell:1844
SLE	Human	10x Genomics	7140	11833	-	GSE162577	-

13 Section 13: Tutorial of PredGCN Webserver

Figure S21, S22, S23, S24, S25, S26, S27

The utilization of the PredGCN webserver can be approached via two distinct pathways: the utilization of USE SAMPLE DATASETS and the option to UPLOAD CUSTOM DATASETS. These divergent mechanisms can be accessed through this specific URL: `http://www.aibio-lab.com/PredGCN/index/`, either by selecting the 'Home' tab or the 'Server' tab, as indicated in Figure S21:

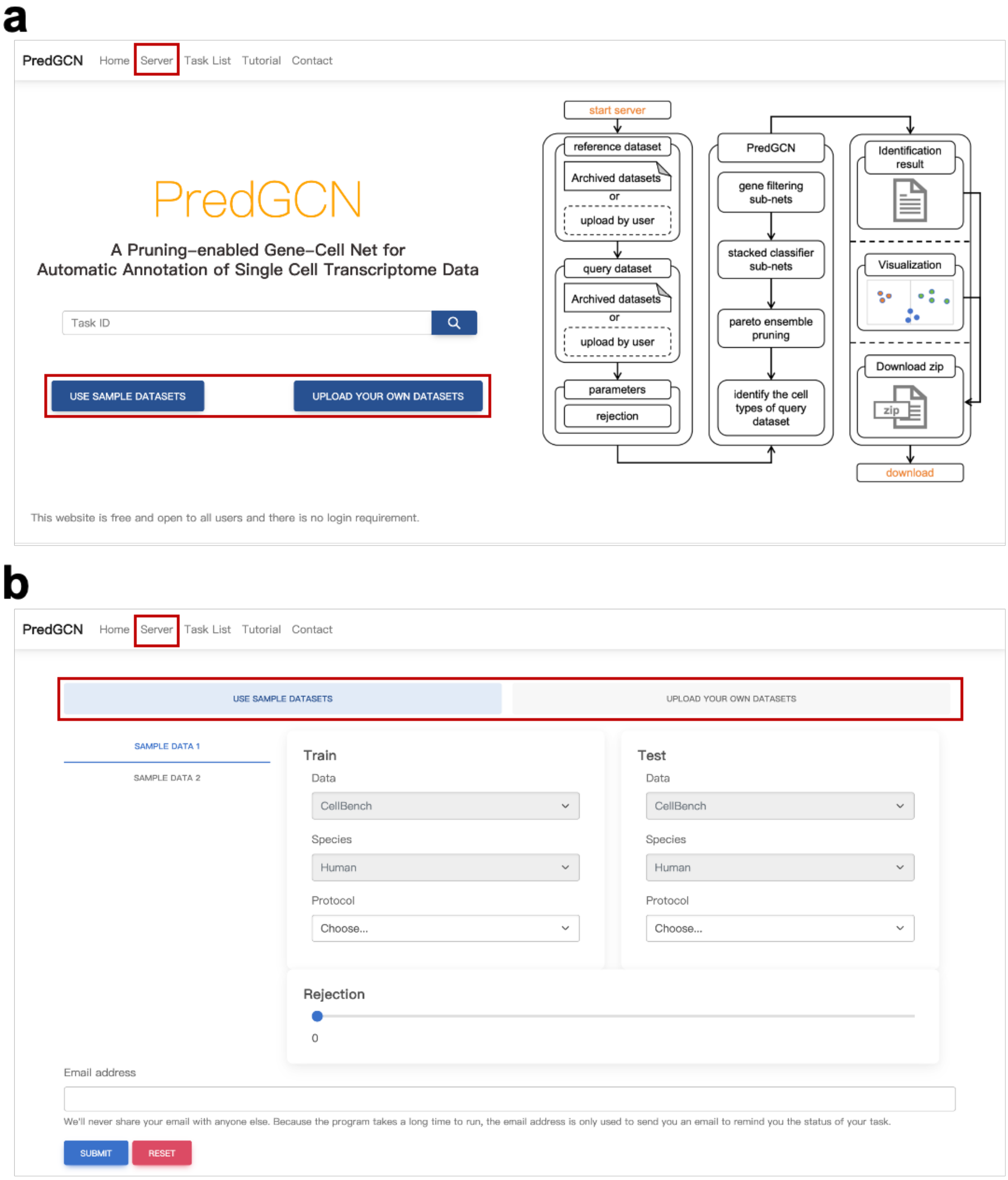

Figure S21: Two ways to use PredGCN webserver, and two access methods to the usages.

Within the confines of the 'USE SAMPLE DATASETS' tab, we offer access to two distinct sample datasets, as depicted in Figure S22. Take Figure S22(a) as the example, users are required to populate the fields encapsulated within the red boundary, with mandatory fields clearly demarcated. Upon completion of these prerequisite fields, users can finalize their task submission by clicking the 'SUBMIT' button.

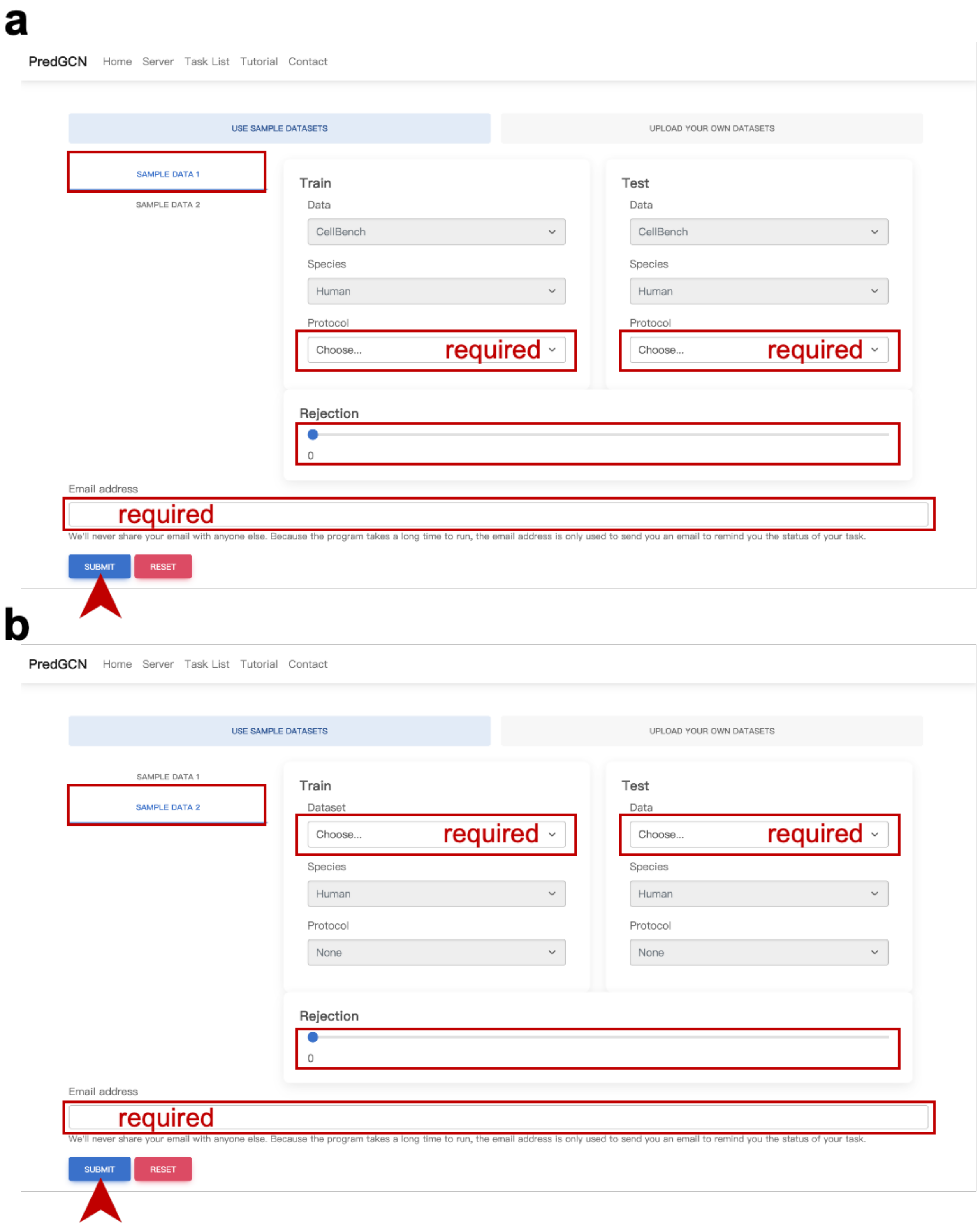

Figure S22: Usages of PredGCN webserver under the USE SAMPLE DATASETS tab.

Within the 'UPLOAD YOUR OWN DATASETS' tab (Figure S23), users have the capacity to upload bespoke train and test datasets, provided they adhere to a predetermined format. Initially, users are required to complete the fields outlined by the red box in Figure S23(a). Subsequently, the datasets are to be uploaded. It is paramount to note that three distinct files necessitate uploading, with specific denominations, namely traindata.csv, trainlabel.csv, and testdata.csv. The formatting for these files should mirror that depicted in Figure S24. As shown in Figure S23(b), users initiate file selection by clicking 'BROWSE', followed by 'UPLOAD' to transmit the files. After uploading files successfully, users are to fill the remaining fields as indicated in Figure S23(c), and then click 'SUBMIT' button to submit

the task.

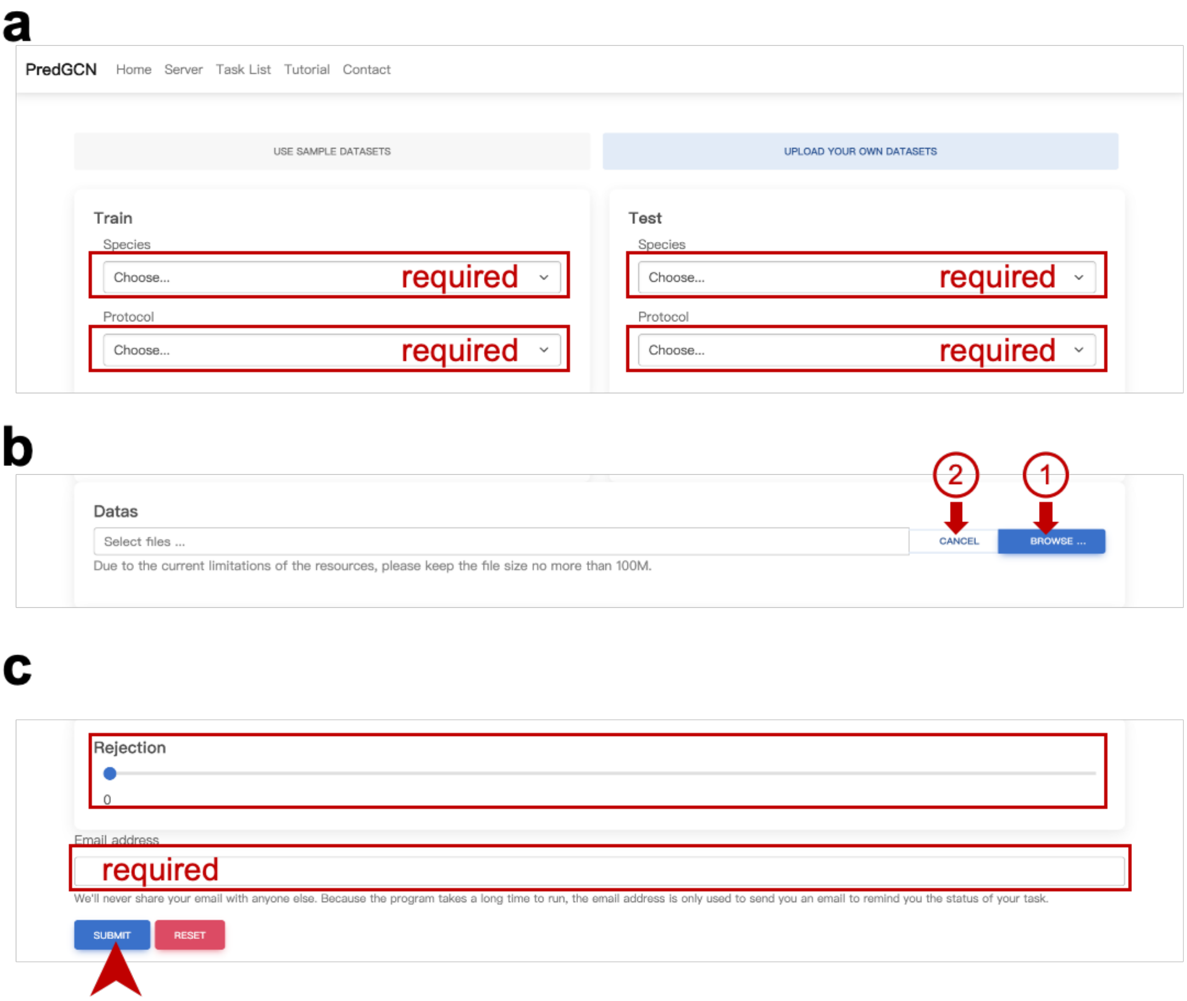

Figure S23: Usages of PredGCN webserver under the UPLOAD YOUR OWN DATASETS tab.

traindata.csv and testdata.csv

	Gene 1	Gene 2	Gene 3	...
Cell 1	...	...	...	...
Cell 2	...	...	...	...
Cell 3	...	...	...	...
...	...	...	...	...

trainlabel.csv

cell.type
label1
label2
label3
...

Figure S24: The format of traindata.csv, trainlabel.csv and testdata.csv.

After submitting your task, a window containing the Task ID will manifest, as depicted in Figure

S25(a). Approximately 3 seconds thereafter, the user interface will autonomously transition to the Task List page, illustrated in Figure S25(b). With the provided Task ID, users can monitor the realtime status of their corresponding task. The details of the corresponding task will be exhibited (FigureS26) by clicking the details button (marked by the red box in Figure S25(b)). The fruition of the task triggers the activation of the 'Download' button, as demonstrated in Figure S26(b), thereby enabling users to download the resultant data. For convenience, users can directly navigate to `http://www.aibio-lab.com/PredGCN/task_list/` to monitor their tasks.

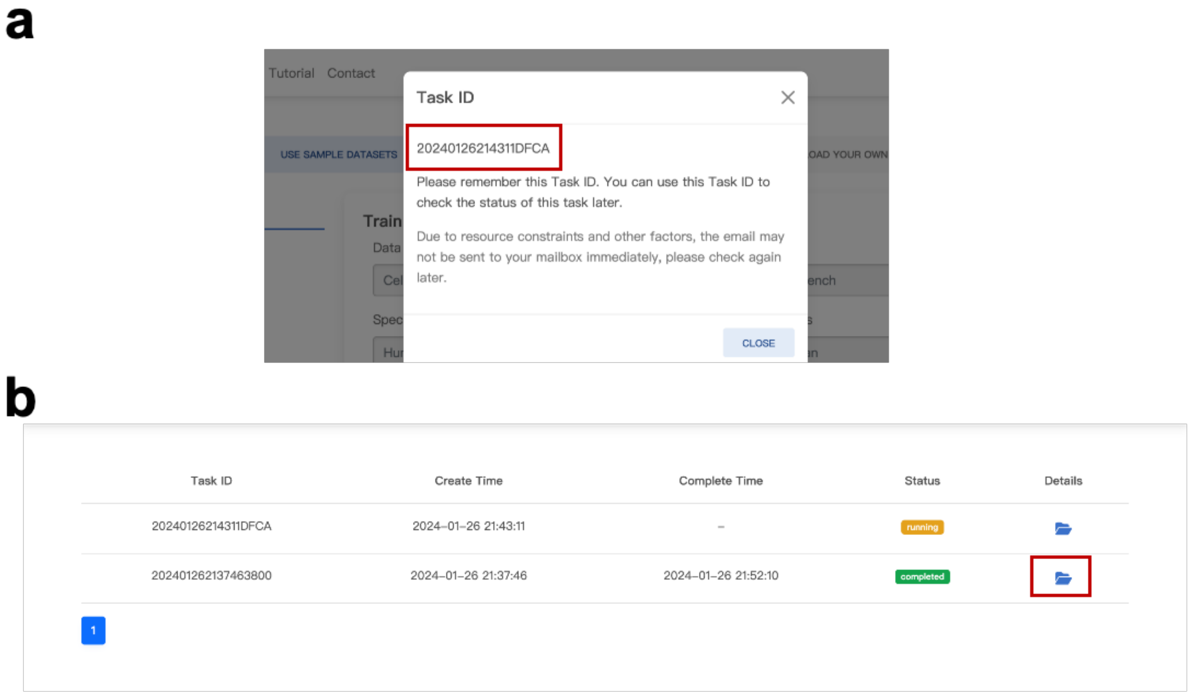

Figure S25: The interface after the task is submitted.

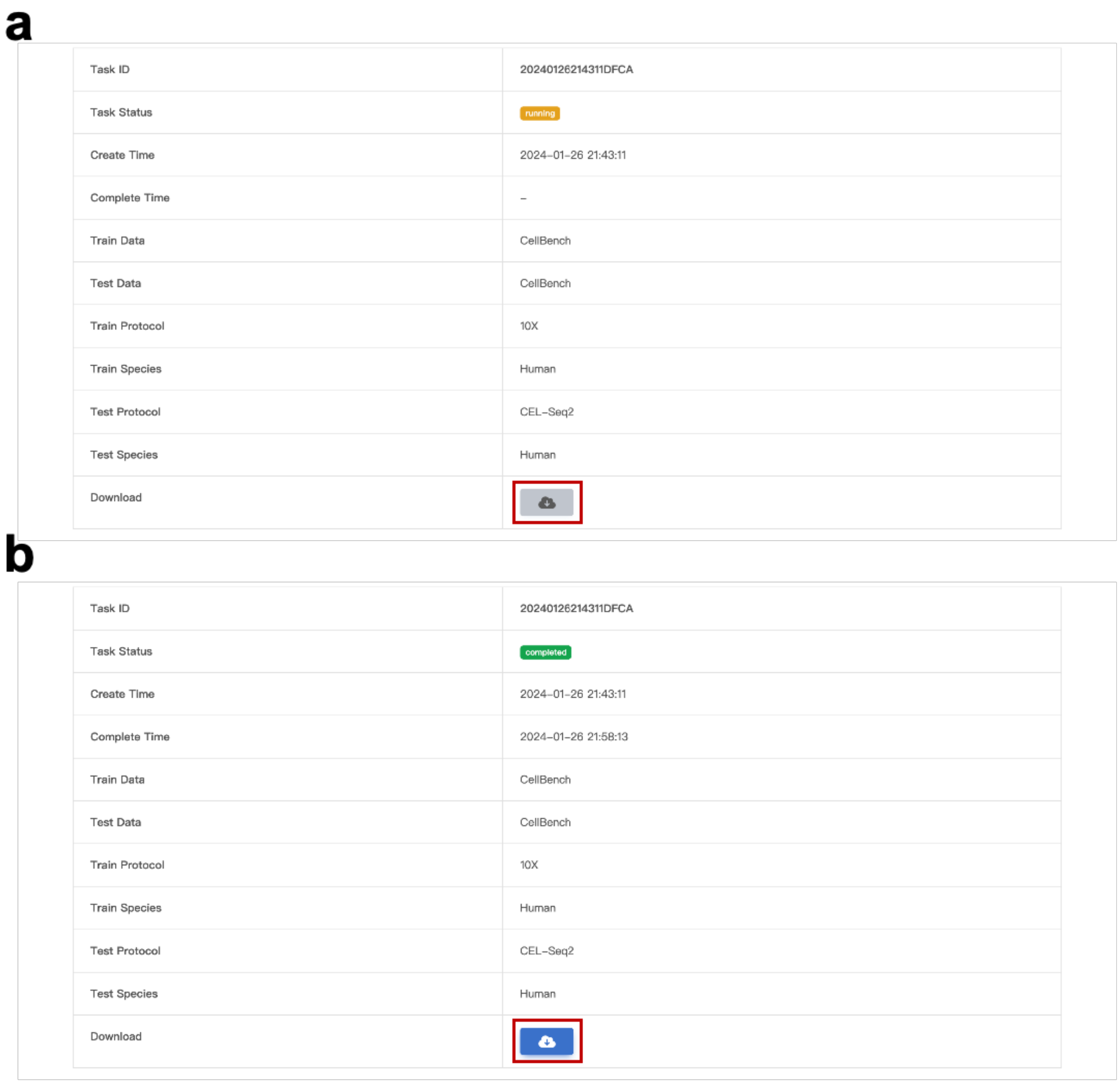

Figure S26: The interface after the task is submitted.

As an alternative provision, we also avail a search feature at `http://www.aibio-lab.com/PredGCN/index/`, as demonstrated in Figure S27(a). Following the task's completion, results can be directly retrieved by utilizing the provided Task ID, as illustrated in Figure S27(b). When the task is completed, users can engage the 'DOWNLOAD" button to acquire the results.

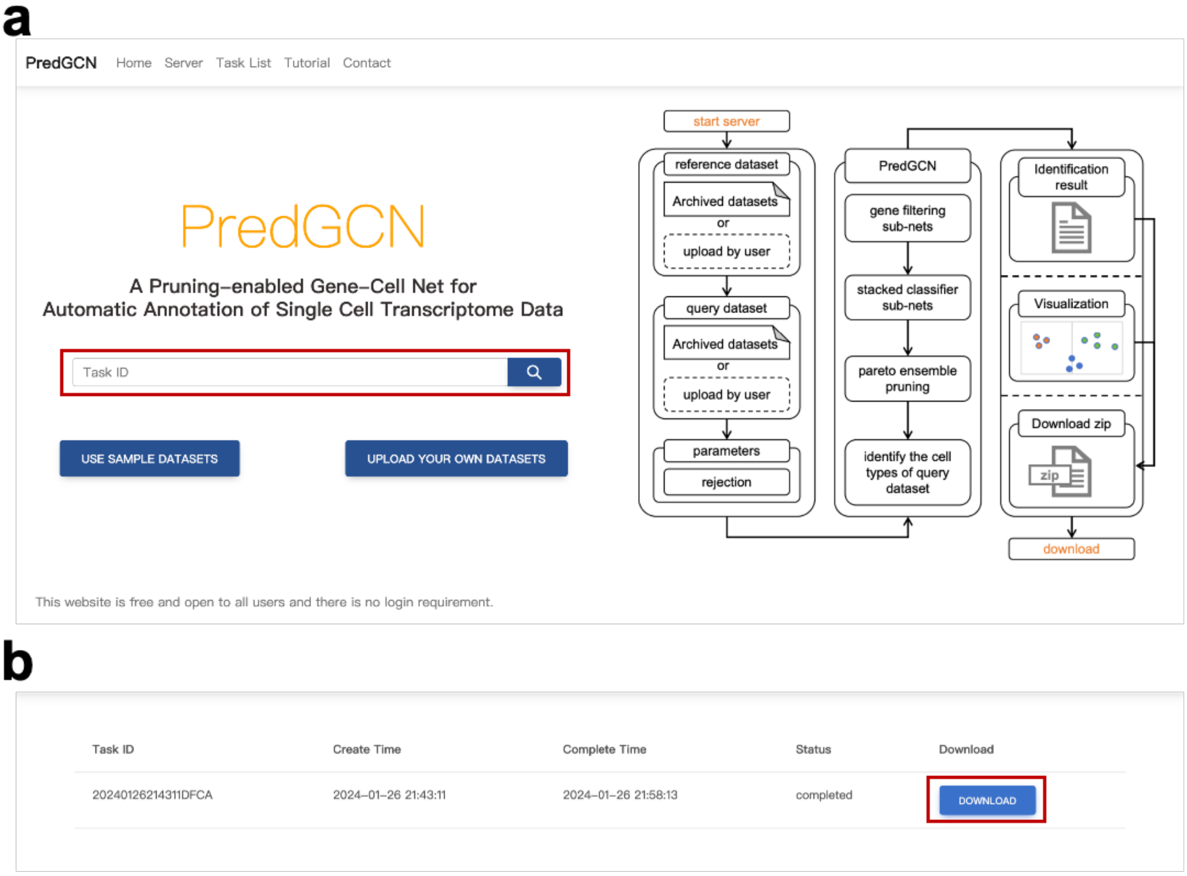

Figure S27: Search task with Task ID.

References

Ding, J. *et al.* (2019). Systematic comparative analysis of single cell rna-sequencing methods. *BioRxiv*, page 632216.

Ma, Y. *et al.* (2020). Integrative differential expression and gene set enrichment analysis using summary statistics for scrna-seq studies. *Nature communications*, **11**(1), 1585.

Pedregosa, F. *et al.* (2011). Scikit-learn: Machine learning in Python. *Journal of Machine Learning Research*, **12**, 2825–2830.

Stoeckius, M. *et al.* (2017). Simultaneous epitope and transcriptome measurement in single cells. *Nature methods*, **14**(9), 865–868.

Szklarczyk, D. *et al.* (2023). The string database in 2023: protein–protein association networks and functional enrichment analyses for any sequenced genome of interest. *Nucleic acids research*, **51**(D1), D638–D646.

Zhou, Q. and Melton, D. A. (2018). Pancreas regeneration. *Nature*, **557**(7705), 351–358.
